# ScaleSpecter: a frequency-aware multi-scale patch framework for robust physiological classification under non-stationarity

**DOI:** 10.3389/fnhum.2026.1856803

**Published:** 2026-07-27

**Authors:** Zhouyang Xu, Hongwei Li, Wenchao Liu, Haifeng Li

**Affiliations:** Faculty of Computing, Harbin Institute of Technology, Harbin, China

**Keywords:** collaborative feature representation, EEG signals, neurodegenerative diseases, phase-amplitude coupling, sensitive pathological features

## Abstract

**Introduction:**

Early detection and intervention for cognitive impairment associated with neurodegenerative diseases are important for slowing disease progression and improving quality of life. Electroencephalography provides high temporal resolution and sensitivity to neural oscillations, making it a promising tool for early disease identification. However, weak and transient pathological abnormalities are often obscured by diffuse, non-stationary low-frequency background rhythms, making robust feature extraction challenging.

**Methods:**

We propose ScaleSpecter, a frequency-aware multiscale patch framework for neurodegenerative disease-related EEG classification. ScaleSpecter first constructs temporal representations at multiple scales to jointly capture transient local abnormalities and long-term rhythmic variations. A lightweight cross-scale attention mechanism then enables interaction between fine-grained temporal tokens and coarse scale-level summaries. Finally, an amplitude-phase-aware spectral modulation module uses learnable complex-valued weights to recalibrate spectral responses and provide frequency-domain guidance for temporal feature aggregation.

**Results:**

Extensive experiments were conducted on three public EEG datasets: the Alzheimer's Disease and Frontotemporal Dementia (ADFTD) dataset, the Alzheimer's Patients' Relatives Association of Valladolid (APAVA) dataset, and the Two Decades-Brainclinics Research Archive for Insights in Neurophysiology (TDBRAIN) database. These datasets cover classification tasks related to Alzheimer's disease, frontotemporal dementia, and Parkinson's disease. ScaleSpecter achieved competitive and generally favorable performance on most key evaluation metrics. The ablation and visualization results further demonstrated the complementary contributions of multiscale temporal modeling, cross-scale interaction, and spectral modulation.

**Discussion:**

The results suggest that integrating frequency-domain guidance with multiscale temporal representations can improve the discriminative capability and robustness of EEG classification under non-stationary conditions. ScaleSpecter provides a potentially generalizable framework for neurodegenerative disease-related physiological signal analysis.

## 1 Introduction

Electroencephalography (EEG) is a non-invasive technique that records brain activity through scalp electrodes. Owing to its high temporal resolution, favorable safety profile, relatively low acquisition cost, and sensitivity to large-scale neural dynamics, EEG has been widely used in cognitive function research and in the auxiliary analysis of neurological disorders, particularly in neurodegenerative disease-related tasks, including Alzheimer's disease (AD), frontotemporal dementia (FTD), and Parkinson's disease (PD) (Wang et al., 2022; Ehteshamzad, 2024).

However, EEG signals are characterized by marked non-stationarity and complex time-frequency structures. In the early stages of neurodegenerative diseases, discriminative pathological patterns do not usually appear as single, stable local morphologies; rather, they are often manifested as subtle and dispersed abnormalities across multiple frequency bands, together with fluctuations between local transient perturbations and long-term background rhythms (Chen et al., 2023). Previous studies have shown that EEG abnormalities in neurodegenerative diseases are often associated with enhanced low-frequency activity, slowing of background rhythms, and disrupted coordination across frequencies (Zheng et al., 2023; Chen et al., 2023; Burelo et al., 2024). In Alzheimer's disease, such alterations have also been reported to be related to neurodegenerative changes in regions such as the hippocampus, entorhinal cortex, posterior cingulate cortex, and precuneus (Ereira et al., 2024; Zhang et al., 2024). Due to inter-individual variability, noise interference, and diffuse background activity, traditional methods often struggle to identify these subtle patterns in a stable manner. Therefore, how to simultaneously characterize short-duration high-frequency perturbations and long-timescale low-frequency rhythmic changes, while maintaining sensitivity to weak pathological cues under strong background fluctuations, remains a critical issue in EEG classification for neurodegenerative disease-related tasks (Zheng et al., 2023; Burelo et al., 2024; Zheng et al., 2025).

**Figure 1:**
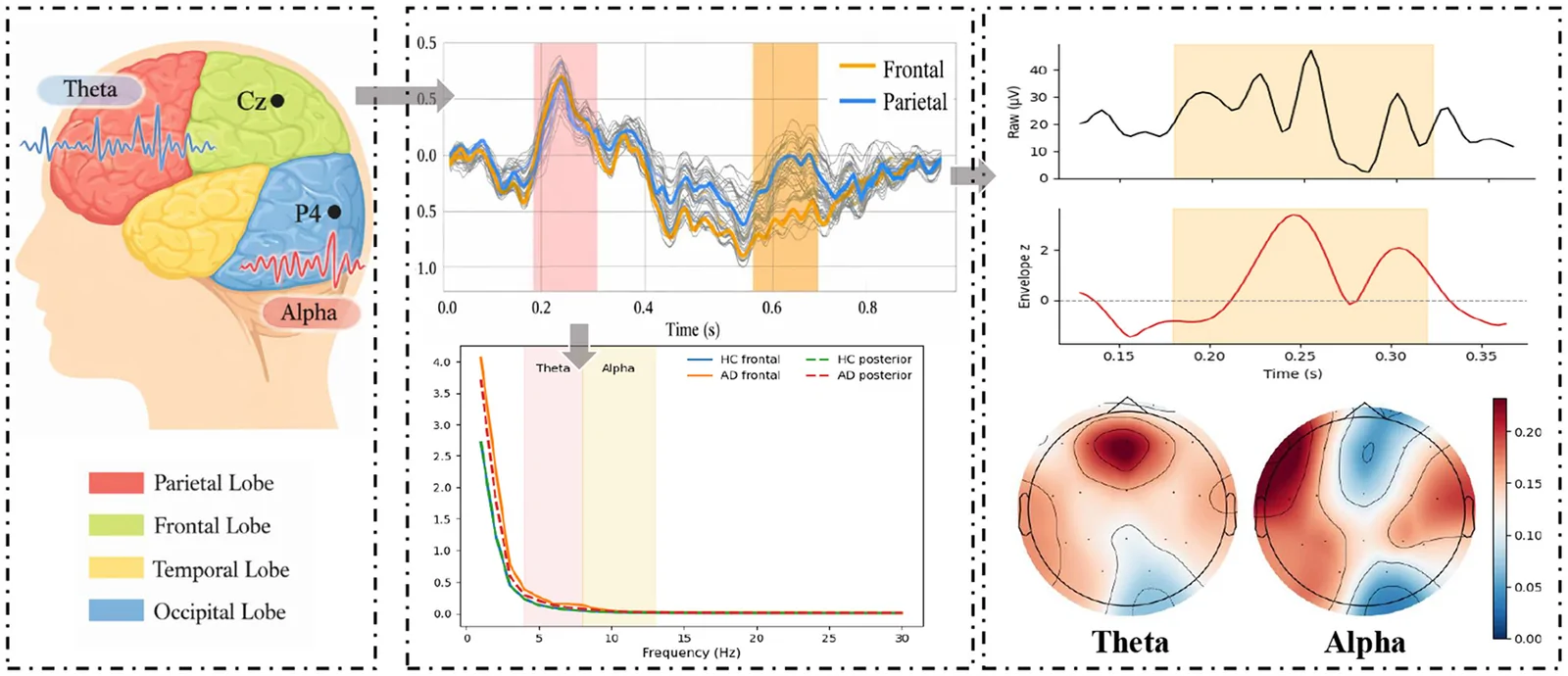
Local short-duration activity and long-timescale rhythmic variations in raw EEG.

As shown in Figure 1, the discriminative features of neurodegenerative disease-related EEG exhibit pronounced cross-scale characteristics. On the one hand, local short-duration fast activity is often buried within complex background fluctuations in a weak and transient form, making it difficult to identify through direct observation in the time domain (Chen et al., 2023). On the other hand, long-timescale low-frequency rhythmic changes are more commonly manifested as spectral slowing trends and rhythm reorganization in frequency bands such as theta and alpha (Zheng et al., 2023; Burelo et al., 2024). Previous clinical and imaging studies have shown that typical AD-related abnormalities often involve the posterior cingulate cortex, precuneus, posterior temporoparietal association cortex, and medial temporal lobe, and are associated with memory impairment, visuospatial integration deficits, and broader cognitive decline (Ereira et al., 2024; Zhang et al., 2024). These observations suggest that disease-related EEG abnormalities are not isolated disturbances confined to a single frequency band, but are more likely reflected as distributed temporal, spectral, and spatial alterations. This also reveals a fundamental challenge faced by existing methods: if only local transient changes are considered, long-timescale rhythmic backgrounds may be overlooked; conversely, if only global spectral statistics are used, local short-duration abnormalities may be obscured (Burelo et al., 2024; Zheng et al., 2025).

Existing studies have mainly addressed this problem from two perspectives. One line of research focuses on multiscale temporal modeling, leveraging the complementarity of representations at different temporal resolutions to characterize complex sequential patterns. Shorter temporal windows are advantageous for preserving local variations and transient perturbations, whereas longer windows are better suited to capturing slow-varying trends and global rhythms. Based on this understanding, related methods typically model local patterns and global structures through multi-granularity segmentation, cross-scale interaction, or hierarchical attention mechanisms. For example, CrossViT (Chen et al., 2021) and CrossFormer (Zhang and Yan, 2022) integrate representations at different granularities through multi-branch architectures or long-short range interaction mechanisms; MViT (Fan et al., 2021) organizes multiscale features using a hierarchical structure; and MedFormer (Wang et al., 2024) further demonstrates that multiscale patch representations and interaction mechanisms can improve the modeling of complex physiological signals. These methods provide an important foundation for representing information across different temporal resolutions.

Another line of research focuses on frequency-domain feature modeling. Because discriminative information in EEG is closely related to rhythmic changes and differences in frequency components, previous studies have introduced complementary cues beyond temporal representations through frequency-domain attention, frequency-band selection, or spectral enhancement, thereby improving the model's ability to perceive spectral structures. For instance, spectral enhancement mechanisms such as FEDformer (Zhou et al., 2022) and SpectralFormer (Hong et al., 2021) have demonstrated the effectiveness of frequency-domain modeling; and KEMed (Yuan et al., 2025) improves the representation of complex patterns by incorporating a knowledge-enhancement strategy. Overall, this line of research further underscores the importance of frequency-domain cues in neurodegenerative disease-related EEG analysis.

Although multiscale temporal modeling and frequency-domain modeling have both been shown to be useful for EEG classification, they are still largely treated as separate or loosely coupled components in existing methods. Multiscale modules mainly organize temporal information according to window sizes or token granularities, whereas frequency-domain modules are often used as parallel feature extractors or auxiliary enhancement branches. For highly non-stationary neurodegenerative disease-related EEG, this separation may be suboptimal. Local pathological transients and long-timescale rhythmic backgrounds are not independent: weak short-duration abnormalities may be masked by dominant low-frequency activity, while global rhythmic slowing may change the context in which local perturbations should be interpreted. Therefore, a key yet insufficiently explored problem is how to allow frequency-domain cues to directly participate in the fusion and calibration of temporal representations across different scales.

To address this issue, we propose ScaleSpecter, a frequency-aware multiscale patch framework for neurodegenerative disease-related EEG classification under non-stationary conditions. ScaleSpecter first constructs heterogeneous temporal tokens through multiscale patch embedding, enabling local transient perturbations and long-timescale rhythmic patterns to be represented within a unified token space. It then employs a lightweight cross-scale attention mechanism to exchange information between fine-grained temporal tokens and coarse scale-level summaries. Finally, instead of treating spectral features as an isolated auxiliary branch, ScaleSpecter introduces an amplitude-phase-aware spectral modulation module, in which learnable complex-valued gates recalibrate spectral responses and provide frequency-domain guidance for temporal feature aggregation. We use the term amplitude-phase-aware modulation to emphasize learnable complex spectral recalibration; the module is not intended to directly estimate classical neuroscientific phase-amplitude coupling indices.

The proposed method was systematically evaluated on three public neurodegenerative disease-related EEG datasets. The experimental results show that ScaleSpecter achieves competitive performance compared with multiple strong baselines under the adopted evaluation protocols. Further ablation experiments and visualization analyses indicate that the combination of multiscale temporal representations and frequency-domain guidance helps enhance the model's discriminative capability and robustness under complex background conditions. The main contributions of this study are summarized as follows:

- We propose a frequency-aware multiscale patch framework for neurodegenerative disease-related EEG classification under non-stationary conditions. By constructing heterogeneous temporal tokens at multiple patch scales, the proposed method jointly represents local transient perturbations and long-timescale rhythmic backgrounds within a unified token space.
- We design a lightweight cross-scale attention mechanism that enables bidirectional interaction between fine-grained temporal tokens and coarse scale-level summaries. This design allows temporal representations at different granularities to be collaboratively calibrated without relying on a heavy multi-branch architecture.
- We introduce an amplitude-phase-aware spectral modulation module based on learnable complex-valued gating. Rather than treating spectral features as an isolated auxiliary branch, the proposed module provides frequency-domain guidance for temporal feature aggregation, improving the model's ability to capture weak pathological patterns under dominant non-stationary backgrounds.
- We validate the effectiveness of ScaleSpecter on three public EEG datasets, spanning classification tasks related to both Alzheimer's disease and Parkinson's disease: ADFTD, TDBrain, and APAVA. The results, together with ablation and visualization analyses, demonstrate the effectiveness of the proposed design under the adopted evaluation protocols.

## 2 Materials and methods

### 2.1 Dataset

We systematically evaluated ScaleSpecter on three public EEG datasets: ADFTD (Dauwels et al., 2010), APAVA (Cecchetti et al., 2021), and TDBrain (Liu et al., 2014). Among them, APAVA is a binary classification task for distinguishing healthy controls (HC) from Alzheimer's disease (AD) samples; ADFTD is a three-class classification task for distinguishing healthy controls (HC), frontotemporal dementia (FTD), and Alzheimer's disease (AD); and TDBrain is a binary classification task for distinguishing healthy controls (HC) from Parkinson's disease (PD)-related samples. All three datasets belong to neurodegenerative disease-related EEG classification scenarios and exhibit pronounced non-stationarity and inter-subject variability, making them well-suited for validating the model's ability to characterize complex pathological rhythmic patterns.

In the preprocessed versions used in this study, APAVA contains 23 subjects, 5,967 samples, and 16 channels; ADFTD contains 88 subjects, 69,752 samples, and 19 channels; and TDBrain contains 72 subjects, 6,240 samples, and 33 channels. To ensure comparability across datasets, all samples were processed into fixed-length EEG segments, with each sample containing 256 time points. In addition, the sampling rates of APAVA and ADFTD were 256 Hz, while the processed version of TDBrain was likewise standardized into input segments of length 256. Because this study focuses on non-stationary EEG classification for neurodegenerative diseases, only EEG-related experimental results are reported in the main text.as summarized in Table 1.

**Table 1:** Dataset information.

Dataset	Subjects	Samples	Classes	Channels	Time points	Sampling rate	Modality
APAVA	23	5,967	2	16	256	256 Hz	EEG
ADFTD	88	69,752	3	19	256	256 Hz	EEG
TDBrain	72	6,240	2	33	256	256 Hz	EEG

To comprehensively evaluate model performance, two commonly used experimental settings were adopted: Subject-Dependent and Subject-Independent. In the Subject-Dependent setting, the training, validation, and test sets were split at the sample level, and therefore samples in different splits could originate from the same group of subjects. In the Subject-Independent setting, the data were divided according to subject identity, thereby ensuring that the training and test sets were strictly independent at the subject level and more accurately reflecting the model's generalization ability to unseen individuals. Given that real-world clinical scenarios place greater emphasis on cross-individual generalization, the Subject-Independent results were treated as the primary results in the analysis. All results are reported as the mean and standard deviation over multiple independent runs.

For the five-fold cross-validation experiments, we adopted a subject-level partition strategy. Specifically, all subjects in each dataset were first partitioned into five non-overlapping outer folds in a class-stratified manner whenever possible. In each round, one outer fold was used as the held-out test set, and the remaining subjects were further divided into training and validation sets at the subject level. This procedure was repeated five times so that each outer fold served as the test set once. All EEG segments from the same subject were assigned to the same split, ensuring that no subject overlap existed among the training, validation, and test sets. The validation set was used for model selection and early stopping, while the final performance was computed on the held-out test fold. The reported results are the mean and standard deviation over the five test folds.

### 2.2 Evaluation

Six evaluation metrics, namely Accuracy, Precision, Recall, F1-score, AUROC, and AUPRC, were adopted to comprehensively assess the model performance. For multiclass tasks, Precision, Recall, and F1-score were calculated using the macro-average scheme to mitigate the influence of class imbalance on the evaluation results. Let there be *K* classes in total, and let the true positives, false positives, and false negatives of class *c* be denoted by *TP*_*c*_, *FP*_*c*_, and *FN*_*c*_, respectively. Let the total number of samples be *N*.

For AUROC and AUPRC, the scores for binary classification tasks are computed directly from the predicted probabilities of the positive class, whereas for multiclass tasks, a one-vs-rest strategy is adopted to compute the areas under the ROC and PR curves for each class separately, followed by macro-averaging across all classes. AUROC measures the overall ability of the model to distinguish between positive and negative samples across different decision thresholds, whereas AUPRC places greater emphasis on the quality of positive-sample identification under class-imbalanced conditions. Therefore, together with metrics such as Accuracy and F1-score, these two measures provide a more comprehensive evaluation of classification performance.

### 2.3 Method

**Figure 2:**
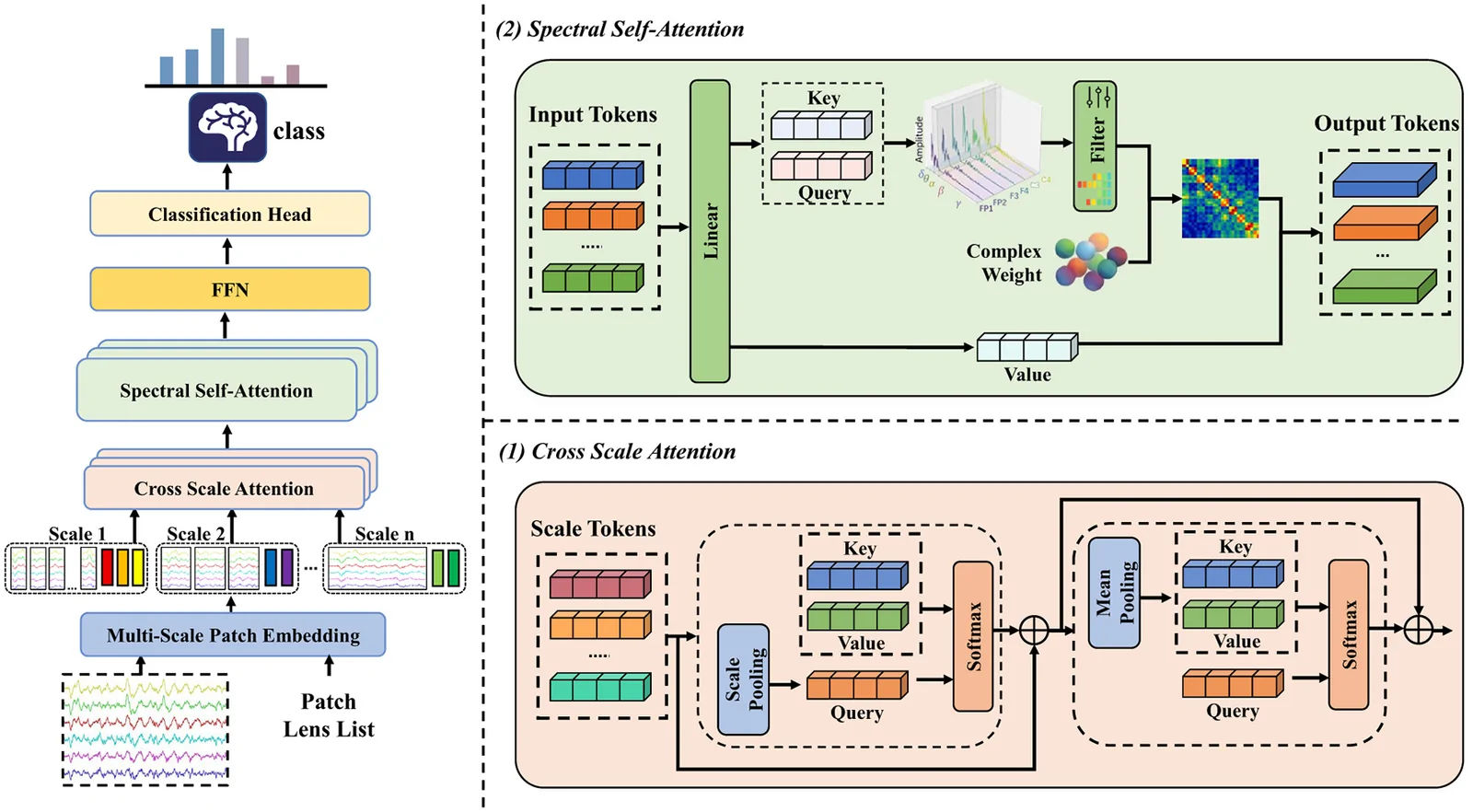
Overall architecture of ScaleSpecter. The model first constructs token representations at different temporal resolutions through multiscale temporal tokenization; it then coordinates coarse-grained and fine-grained information via cross-scale context calibration; finally, an amplitude-phase-aware frequency-domain guidance mechanism is employed to provide auxiliary cues for temporal aggregation, and the final classification result is produced.

This section presents the overall modeling pipeline of ScaleSpecter. To address the non-stationary characteristics of neurodegenerative disease-related EEG, the model is designed as a continuous information flow. First, a unified temporal representation is constructed at different temporal resolutions through multiscale temporal tokenization. Second, a cross-scale context calibration module is employed to coordinate coarse-grained and fine-grained information. Finally, an amplitude-phase-aware frequency-domain guidance mechanism is introduced to provide additional frequency-domain evidence for cross-scale temporal fusion, thereby enhancing the model's ability to capture the coexistence of local pathological perturbations and global background rhythms. The overall architecture is illustrated in Figure 2.

#### 2.3.1 Multi-scale patch encoding and cross-scale aggregation

**Figure 3:**
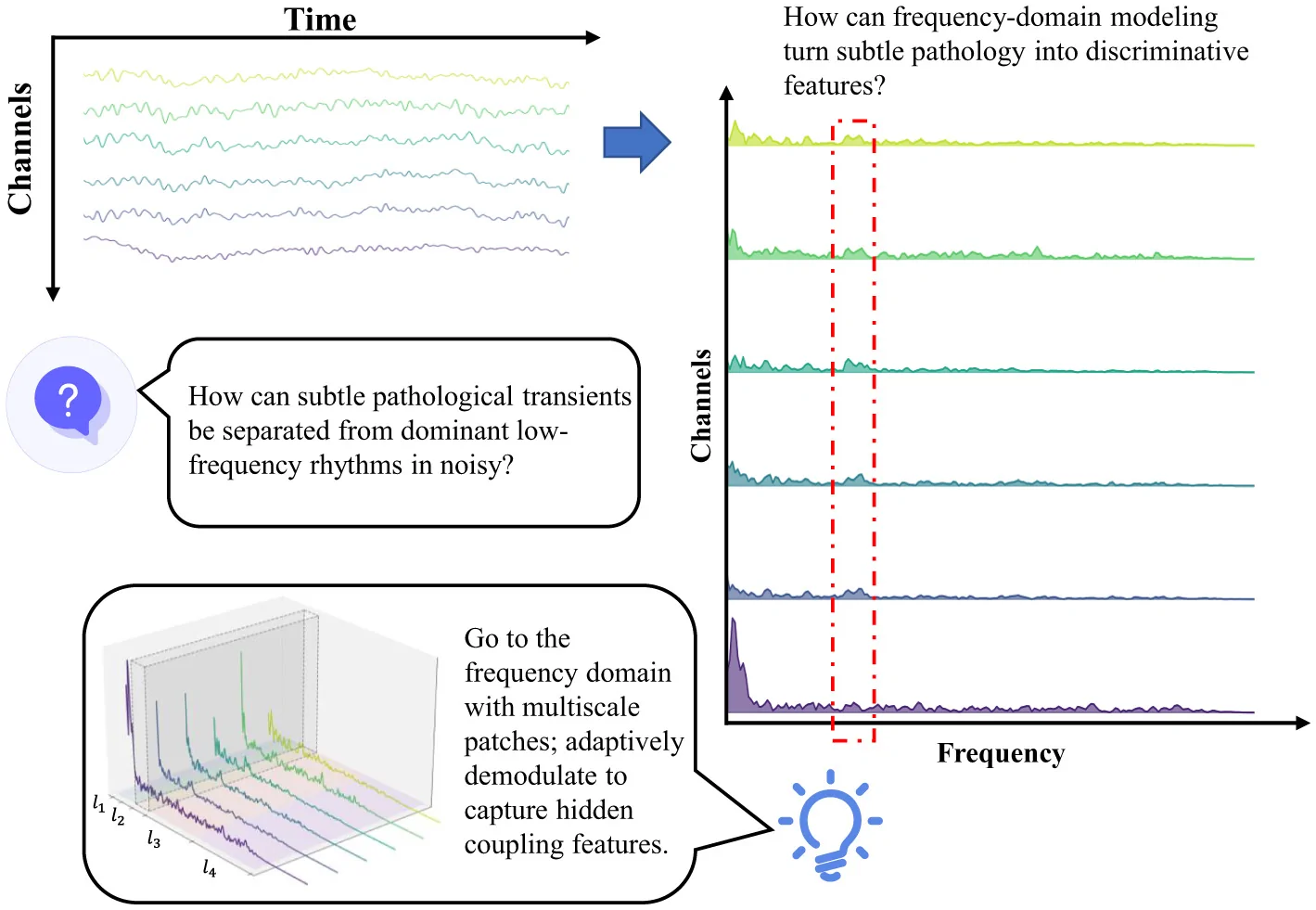
Illustration of the feature-masking phenomenon in raw signals, the enhancement effect of adaptive demodulation on hidden frequency-domain features, and how multiscale patches overcome the resolution bottleneck.

As illustrated in Figure 3. The discriminative patterns of neurodegenerative disease-related EEG are typically distributed across different temporal scales: slow-varying rhythmic backgrounds reflect global changes over longer time spans, whereas local transient perturbations are more often manifested as short-duration abnormalities. To preserve both types of information within a unified representation space, multiscale patch representations are adopted as the basis for temporal encoding, and complementary temporal structures are extracted at different temporal granularities during the input stage.

Specifically, the input sequence is partitioned using multiple temporal window sizes, and the patches obtained at different scales are projected into a unified feature space and then concatenated to form a multiscale token representation. This design enables the model to obtain candidate representations of both local variations and global trends at the early stage of encoding, while providing a unified input for subsequent cross-scale aggregation. On this basis, the amplitude-phase information modeled by the frequency-domain branch can further participate in the fusion of representations across different scales.

Let the input EEG time series be denoted as *X* ∈ ℝ^*B*×*T*×*C*^, where *B* denotes the batch size, *T* the number of time steps, and *C* the number of electrode channels. The multiscale slicing window set is defined as $P=\left\{p_{1},p_{2},\ldots ,p_{M}\right\}$, where each scale *p*_*m*_ represents a sampling window length. To ensure divisibility along the temporal axis, each sequence is zero-padded at the end for scale *p*_*m*_, and the padded temporal length is denoted as ${\bar{T}}_{m}$, satisfying ${\bar{T}}_{m}\;\mathrm{mod}\;p_{m}=0$. Accordingly, the number of patches at scale *p*_*m*_ is denoted by $n_{m}={\bar{T}}_{m}/p_{m}$, and a length-aligned tensor ${\bar{X}}^{\left(m\right)}\in {\mathbb{R}}^{B\times {\bar{T}}_{m}\times C}$ is constructed and then flattened into a patch sequence, as formulated in Equation 1:


$$\begin{array}{l} P^{\left(m\right)}=\text{reshape}\left({\bar{X}}^{\left(m\right)},B,n_{m},p_{m}\times C\right)\in {\mathbb{R}}^{B\times n_{m}\times \left(p_{m}C\right)}. \end{array}$$ (1)

Unlike conventional single-channel patches, each patch in the proposed method encapsulates the EEG signal variations of all channels within the same temporal window, thereby enhancing its ability to model spatially coordinated features. Subsequently, an independent linear transformation matrix $W_{p_{m}}\in {\mathbb{R}}^{\left(p_{m}C\right)\times D}$ is applied to the patch sequence *P*^(*m*)^ at each scale to project it into a global feature space with a unified dimension *D*, and the representations from all scales are concatenated in the order from long windows to short windows all multiscale representations are defined in in Equations 2 and 3, respectively:


$$\begin{array}{l} Z^{\left(m\right)}=P^{\left(m\right)}W_{p_{m}}\in {\mathbb{R}}^{B\times n_{m}\times D} \end{array}$$ (2)

$$\begin{array}{l} Z=\left[Z^{\left(1\right)},Z^{\left(2\right)},\ldots ,Z^{\left(M\right)}\right]\in {\mathbb{R}}^{B\times \sum_{m=1}^{M}n_{m}\times D} \end{array}$$ (3)

The concatenated token sequence preserves both multichannel coordinated structures and information at multiple temporal granularities, enabling the model to capture both global rhythmic trends and transient pathological fluctuations in EEG within a single attention layer, thereby avoiding the efficiency bottleneck associated with the deep stacked architectures used in conventional methods. In addition, non-overlapping sinusoidal positional encodings are adopted at each temporal scale to distinguish the positional information of different tokens, thereby explicitly providing the model with scale-aware and temporal contextual identifiers and enhancing its ability to discriminate structural patterns.

**Figure 4:**
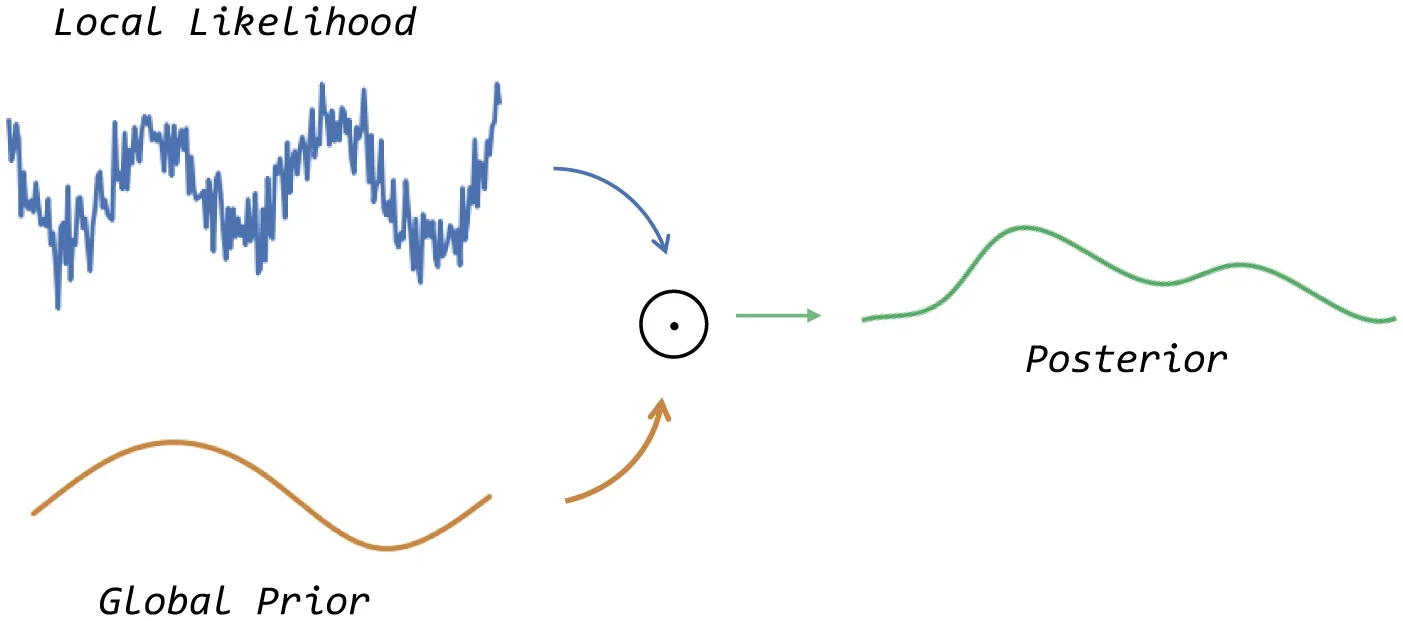
Schematic illustration of cross-scale feature decoupling and reconstruction. **(Left)** Coarse-grained guidance stage. The model first extracts a smooth low-frequency trend (blue curve) from the raw noisy signal to establish global contextual dependencies and suppress high-frequency interference. **(Right)** Detail-focused stage. During the modeling of high-frequency textures (orange curve), the trend extracted in the first stage is used as prior guidance, enabling the model to focus on capturing fine-grained local oscillatory patterns.

The discriminative patterns of neurodegenerative disease-related EEG typically span multiple temporal scales, comprising both slowly varying rhythmic backgrounds and local transient perturbations. To characterize both types of information simultaneously, a lightweight cross-scale aggregation module is built upon the multiscale representations to enable a two-stage interaction between coarse-grained and fine-grained tokens. Unlike approaches that establish symmetric interactions directly between representations at different granularities, CSA uses scale summaries as an intermediary: global context is first transmitted to local representations, after which the resulting local responses are used to update the scale-level representations in return, thereby forming an aggregation pathway that proceeds from coarse to fine and then from fine to coarse. As shown in Figure 4, this design allows the cross-scale fusion process to be further organized as a representation update procedure that can be modulated by frequency-domain information.

Let the multiscale patch token sequence be denoted as *Z* ∈ ℝ^*B*×*N*×*D*^, where *B* denotes the batch size, *N* denotes the total number of tokens across all scales, and *D* the feature dimension. These tokens can be partitioned into *M* groups, $\left\{S_{1},S_{2},\ldots ,S_{M}\right\}$, according to their corresponding scales, where the *m*-th scale $S_{m}$ contains *n*_*m*_ tokens and satisfies $\overset{M}{\underset{m=1}{\sum }}n_{m}=N$.

First, to capture the global statistical characteristics at different scales, average pooling is performed over the tokens within each scale to generate scale summaries as defined in Equation 4:


$$\begin{array}{l} q_{m}=\frac{1}{n_{m}}\underset{z_{i}\in S_{m}}{\sum }z_{i},\;Q=\text{Stack}\left(\left[q_{1},\ldots ,q_{M}\right]\right)\in {\mathbb{R}}^{B\times M\times D} \end{array}$$ (4)

With the scale summaries *Q* used as the Query and the full set of fine-grained tokens *Z* used as the Key and Value, multi-head attention (MHA) is performed to aggregate global contextual information according to Equation 5:


$$\begin{array}{l} \hat{Q}=\text{MHA}\left(Q,Z,Z\right) \end{array}$$ (5)

The attention output $\hat{Q}\in {\mathbb{R}}^{B\times M\times D}$ contains scale-level features enhanced by information aggregated from all tokens. To feed this coarse-grained information back to the fine-grained tokens, each scale summary ${\hat{q}}_{m}$ in $\hat{Q}$ is repeated *n*_*m*_ times along the sequence dimension so that its shape is aligned with that of the original input *Z*, and the resulting tensor is denoted by $\text{Expand}\left(\hat{Q}\right)\in {\mathbb{R}}^{B\times N\times D}$. Subsequently, a residual connection is applied, yielding the features updated after the first stage, *Z*′ as defined in Equation 6:


$$\begin{array}{l} Z^{\prime }=Z+\text{Expand}\left(\hat{Q}\right) \end{array}$$ (6)

This process allows the statistical patterns of global low-frequency rhythms to modulate the representations of high-frequency tokens in a top-down manner, thereby improving their discriminative capability under complex background conditions.

Subsequently, to strengthen the feedback of fine-grained differences to coarse-scale representations, the roles of the Query and Key/Value are exchanged. First, new scale summaries are recomputed from the updated *Z*′ and used as the Key and Value as defined in Equation 7:


$$\begin{array}{r} k_{m}^{\prime }=v_{m}^{\prime }=\frac{1}{n_{m}}\underset{z_{i}^{\prime }\in S_{m}}{\sum }z_{i}^{\prime },\\ K^{\prime }=V^{\prime }=\text{Stack}\left(\left[k_{1}^{\prime },\ldots ,k_{M}^{\prime }\right]\right)\in {\mathbb{R}}^{B\times M\times D} \end{array}$$ (7)

Next, with the fine-grained tokens *Z*′ used as the Query and the new scale summaries *K*′, *V*′ used as the Key and Value, the attention operation is performed according to Equation 8:


$$\begin{array}{l} O_{2}=\text{MHA}\left(Z^{\prime },K^{\prime },V^{\prime }\right) \end{array}$$ (8)

At this stage, the output $O_{2}\in {\mathbb{R}}^{B\times N\times D}$ has the same shape as *Z*′, and a residual connection can therefore be directly applied to obtain the final output defined in Equation 9:


$$\begin{array}{l} Z_{\text{CSA}}=Z^{\prime }+O_{2} \end{array}$$ (9)

Notably, the two stages of MHA share the same set of learnable parameters, forming a tightly coupled closed loop of top-down modulation and bottom-up feedback. This mechanism enables deep interaction among EEG features at different granularities within a single forward pass, while maintaining an overall computational complexity of O(MND), which is substantially lower than the $O\left(N^{2}D\right)$ complexity of standard global attention, since the number of scales satisfies *M* ≪ *N*. In addition, all tokens are modeled within a unified sequence without requiring a branched architecture, thereby effectively improving the efficiency of collaborative feature representation.

#### 2.3.2 Spectral self-attention

To further refine the cross-scale calibrated temporal tokens, we introduce a Spectral Self-Attention (SSA) module. SSA provides frequency-domain guidance through learnable amplitude-phase-aware spectral modulation. This module is inspired by amplitude-phase interactions in the spectral domain, but it is not intended to directly estimate classical neuroscientific phase-amplitude coupling indices.

**Algorithm 1:**
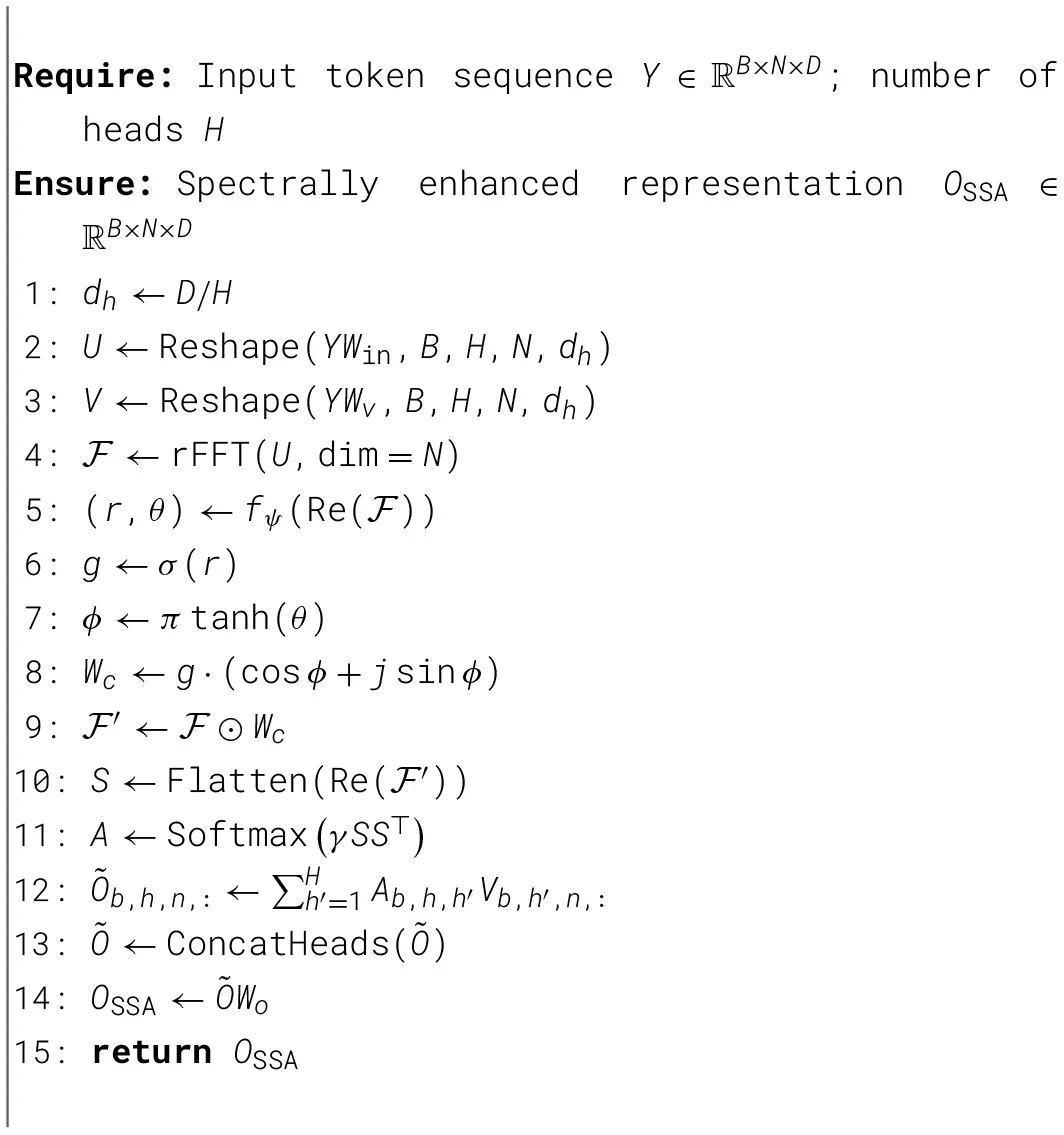
Spectral self-attention (SSA Module).

Let the input of an SSA layer be *Y* ∈ ℝ^*B*×*N*×*D*^, where *N* is the number of tokens and *D* is the embedding dimension. The input is first projected and split into *H* heads as defined in Equations 10 and 11:


$$\begin{array}{l} U=\text{Reshape}\left(YW_{\text{in}},B,H,N,d_{h}\right),\;V=\text{Reshape}\left(YW_{v},B,H,N,d_{h}\right), \end{array}$$ (10)

$$\begin{array}{l} U,V\in {\mathbb{R}}^{B\times H\times N\times d_{h}},\;d_{h}=D/H \end{array}$$ (11)

A real-valued FFT is then applied along the token dimension according to Equation 12:


$$\begin{array}{l} F=\text{rFFT}\left(U,\text{dim}=N\right)\in {\mathbb{C}}^{B\times H\times N_{f}\times d_{h}},\;N_{f}=\left\lfloor \frac{N}{2}\right\rfloor +1 \end{array}$$ (12)

To adaptively modulate the spectral response, a lightweight filter generator *f*_ψ_(·) produces the amplitude and phase gates from the real part of the spectrum according to Equation 13:


$$\begin{array}{l} \left[r,\theta \right]=f_{\psi }\left(\text{Re}\left(F\right)\right),\;g=\sigma \left(r\right),\;\phi =\pi \tanh\left(\theta \right) \end{array}$$ (13)

The complex spectral weight and the modulated spectrum are then computed according to Equation 14:


$$\begin{array}{l} W_{c}=ge^{j\phi }=g\left(\cos\phi +j\sin\phi \right),\;F^{\prime }=F⊙W_{c} \end{array}$$ (14)

Since the modulation is performed by complex-valued multiplication, both the real and imaginary parts of F participate in spectral filtering.

After modulation, the real part of $F^{\prime }$ is flattened within each head to construct spectral descriptors defined in Equation 15:


$$\begin{array}{l} S_{h}=\text{vec}\left(\text{Re}\left(F_{h}^{\prime }\right)\right),\;S\in {\mathbb{R}}^{B\times H\times \left(N_{f}d_{h}\right)} \end{array}$$ (15)

The head-wise spectral attention is computed as The head-wise spectral attention is calculated using Equation 16:


$$\begin{array}{l} A=\text{softmax}\left(\gamma SS^{⊤}\right)\in {\mathbb{R}}^{B\times H\times H}, \end{array}$$ (16)

where γ is a learnable scaling coefficient. The value tensor is obtained from the time-domain projection *V*, and the output of each head is according to Equation 17:


$$\begin{array}{l} O_{b,h,n,:}=\overset{H}{\underset{h^{\prime }=1}{\sum }}A_{b,h,h^{\prime }}V_{b,h^{\prime },n,:} \end{array}$$ (17)

Finally, the heads are merged and projected back to the model dimension using Equation 18:


$$\begin{array}{l} \text{SSA}\left(Y\right)=\text{Merge}\left(O\right)W_{o} \end{array}$$ (18)

The SSA module is used in a standard pre-normalization Transformer block as defined in Equation 19:


$$\begin{array}{l} Y^{\prime }=Y+\text{SSA}\left(\text{LN}\left(Y\right)\right) \end{array}$$ (19)

The Value is obtained from the time-domain token representation through a linear projection, and the final output is produced by aggregating the Value features according to the learned head-wise spectral attention.

The final output *O*_SSA_ represents the features refined by amplitude-phase-aware spectral modulation and head-wise spectral interaction. Given an input *Y* ∈ ℝ^*B*×*N*×*D*^, where *B*, *N*, and *D* denote the batch size, token length, and feature dimension, respectively, SSA first projects the input into *H* heads with head dimension *d*_*h*_ = *D*/*H*. It then applies rFFT along the token dimension, yielding *N*_*f*_ = ⌊*N*/2⌋ + 1 frequency components.

The input and output linear projections contribute $O\left(BND^{2}\right)$, while the rFFT operation contributes O(BDNlogN). The spectral gate generator is applied to each frequency component within each head and introduces $O\left(BHN_{f}d_{h}^{2}\right)$. The head-wise spectral attention requires $O\left(BH^{2}N_{f}d_{h}\right)$ to compute spectral similarities across heads, and the corresponding value aggregation costs $O\left(BH^{2}Nd_{h}\right)$. Therefore, the overall complexity of SSA is given in Equation 20:


$$\begin{array}{l} O\left(BND^{2}+BDN\log N+BHN_{f}d_{h}^{2}+BH^{2}N_{f}d_{h}+BH^{2}Nd_{h}\right). \end{array}$$ (20)

Since *N*_*f*_ ≈ *N*/2 and *d*_*h*_ = *D*/*H*, this can be rewritten as shown in Equation 21:


$$\begin{array}{l} O\left(BND^{2}+BDN\log N+\frac{BND^{2}}{H}+BNDH\right). \end{array}$$ (21)

Because $\frac{BND^{2}}{H}$ is dominated by the projection cost $O\left(BND^{2}\right)$, the overall complexity can be further summarized as as shown in Equation 22:


$$\begin{array}{l} O\left(BND^{2}+BDN\log N+BNDH\right). \end{array}$$ (22)

Compared with standard global self-attention, which requires $O\left(BND^{2}+BN^{2}D\right)$ when including linear projections, SSA replaces quadratic token-wise interactions with head-wise spectral interactions. Since *H* ≪ *N* in practice, SSA avoids the $O\left(BN^{2}D\right)$ token-interaction cost while retaining frequency-domain spectral modulation and temporal value aggregation.

## 3 Results

### 3.1 Baseline methods

To thoroughly validate the effectiveness of ScaleSpecter, a diverse set of representative time-series modeling methods was selected for comparison, including EEGNet (Lawhern et al., 2018), EEGInception (Santamaria-Vazquez et al., 2020), EEGConformer (Song et al., 2022), GPT4TS (Yuan et al., 2025), CrossGNN (Huang et al., 2023), FEDformer (Zhou et al., 2022), FourierGNN (Yi et al., 2023), iTransformer (Liu et al., 2024b), PatchTST (Nie et al., 2023), and TodyNet (Liu et al., 2024a). In addition, two methods specifically designed for medical time series were included: MedFormer (Wang et al., 2024) and KEMed (Yuan et al., 2025).

### 3.2 Implementation details

All experiments were conducted on a single NVIDIA RTX 4090 GPU. In the main fixed-split experiments, all reproduced methods were evaluated over five independent runs, with random seeds set to {41, …, 45} for model initialization and training. The reported results are the mean and standard deviation over these five runs. In addition, subject-level five-fold cross-validation was conducted as a supplementary robustness analysis, where the mean and standard deviation were computed over the five subject-level test folds. The evaluation metrics included Accuracy, Precision, Recall, F1-score, AUROC, and AUPRC. ScaleSpecter was implemented with a 6-layer encoder, with the feature dimension set to *D* = 128 and the hidden dimension of the feed-forward network set to 256.

For fair comparison, all baseline methods with publicly available implementations were reproduced using their official code. Following the unified experimental protocol of Medformer, the optimizer, learning rate, maximum number of epochs, early-stopping strategy, batch size, input length, and evaluation metrics were kept consistent across reproduced methods unless otherwise specified by the official implementation. For baseline methods, the officially recommended hyperparameter settings or the best settings reported in the corresponding papers were adopted. When dataset-specific configurations were not provided, we used the official default settings and adjusted only the input/output dimensions to match the EEG classification tasks. All reproduced methods were evaluated under the same training, validation, and test splits/folds, and no test-set information was used for model selection or hyperparameter tuning.

KEMed and GPT4TS do not provide publicly available source code or complete reproducible configurations. Therefore, direct reproduction was not feasible. The available KEMed-related results were taken from the KEMed paper when the same dataset and evaluation protocol were reported, while unavailable entries are marked as “–”. These literature-reported results are included only as reference values and are not used in runtime, memory, or statistical significance analyses.

Unless otherwise specified by the official implementation, all reproduced methods were trained using the Adam optimizer with a learning rate of 1 × 10^−4^, a maximum of 100 training epochs, and an early stopping strategy with a patience of 10 based on the validation performance. The batch sizes for the three EEG datasets were set to 32 for APAVA, 32 for TDBrain, and 128 for ADFTD, respectively. For ScaleSpecter, only two model-specific hyperparameters, namely the multiscale patch-length list and dropout rate, were selected using the validation set. The candidate patch lengths were selected from {2, 4, 8, 12, 16, 24, 32} to cover both short transient patterns and longer rhythmic backgrounds, and their effect was further analyzed in the patch-length sensitivity study. The final patch configurations were set as follows: *P*_ADFTD_ = {2, 16, 24}, *P*_APAVA_ = {2, 16, 24, 24}, and *P*_TDBrain_ = {8, 16, 32, 32}. The test set was used only for final evaluation.

Following the unified experimental protocol of Medformer, the optimizer, learning rate, maximum number of epochs, early-stopping strategy, batch size, input length, and evaluation metrics were kept consistent across reproduced methods unless otherwise specified by the official implementation. The unified training settings and ScaleSpecter-specific model parameters are summarized in Table 2.

**Table 2:** Unified training settings and ScaleSpecter-specific model parameters.

Parameter	Value
Platform	NVIDIA RTX 4090
Framework	PyTorch 1.12.1
CUDA version	11.3
Optimizer	Adam
Learning rate	1 × 10^−4^
Maximum epochs	100
Early stopping patience	10
Independent runs	5
Random seeds	41–45
Input length	256
Encoder layers	6
Embedding dimension *D*	128
FFN hidden dimension	256
Batch size	32 (APAVA), 32 (TDBrain), 128 (ADFTD)
Patch lengths	APAVA: {2, 16, 24, 24}; ADFTD: {2, 16, 24}; TDBrain: {8, 16, 32, 32}
Dropout	APAVA: 0.35; ADFTD: 0.1; TDBrain: 0.1

### 3.3 Results of subject-dependent

Table 3 presents the experimental results on the reported datasets under the Subject-Dependent setting. As shown in the table, ScaleSpecter achieves the best numerical performance on most evaluation metrics and maintains competitive results compared with multiple strong baselines. Meanwhile, several baseline methods also exhibit strong performance under this setting, suggesting that when the training and test sets contain samples from the same subjects, the data indeed include relatively stable and learnable disease-related patterns. On this basis, ScaleSpecter still obtains further gains, indicating that it possesses a strong capability for modeling discriminative features in neurodegenerative disease-related EEG.

**Table 3:** Results of Subject-Dependent setting on APAVA and ADFTD.

Datasets	Models	Acc (%)	Prec (%)	Rec (%)	F1 (%)	AUROC	AUPRC
**APAVA**	ScaleSpecter	98.34 ± 0.25	98.34 ± 0.20	98.27 ± 0.26	98.27 ± 0.26	99.90 ± 0.02	99.80 ± 0.04
	KEMed	98.29 ± 0.28	98.27 ± 0.32	98.17 ± 0.30	98.22 ± 0.29	99.85 ± 0.04	99.82 ± 0.06
	GPT4TS	98.09 ± 0.32	98.14 ± 0.16	97.89 ± 0.50	98.00 ± 0.34	99.81 ± 0.01	99.83 ± 0.01
	Medformer	96.26 ± 0.34	96.88 ± 0.30	95.44 ± 0.43	96.05 ± 0.37	99.69 ± 0.10	99.70 ± 0.10
	iTransformer	91.34 ± 0.37	90.99 ± 0.26	91.03 ± 0.71	90.97 ± 0.44	97.04 ± 0.08	96.49 ± 0.13
	PatchTST	94.22 ± 0.70	94.08 ± 0.77	93.91 ± 0.79	93.96 ± 0.73	98.83 ± 0.24	98.77 ± 0.26
	FEDformer	92.19 ± 0.44	92.29 ± 0.48	91.37 ± 0.47	91.77 ± 0.47	98.05 ± 0.27	98.02 ± 0.28
	CrossGNN	96.11 ± 0.37	95.97 ± 0.44	95.93 ± 0.34	95.95 ± 0.38	99.23 ± 0.13	99.19 ± 0.16
	FourierGNN	67.09 ± 1.27	65.58 ± 1.41	64.97 ± 0.99	65.12 ± 1.02	71.36 ± 1.50	69.77 ± 1.69
	TodyNet	96.63 ± 0.43	96.82 ± 0.40	96.73 ± 0.50	96.78 ± 0.45	99.53 ± 0.04	99.53 ± 0.04
**ADFTD**	ScaleSpecter	98.09 ± 0.19	97.98 ± 0.20	98.01 ± 0.20	98.00 ± 0.20	99.90 ± 0.02	99.80 ± 0.04
	KEMed	−	−	−	−	−	−
	GPT4TS	−	−	−	−	−	−
	Medformer	97.62 ± 0.34	97.53 ± 0.33	97.48 ± 0.40	97.50 ± 0.36	99.83 ± 0.05	99.62 ± 0.12
	iTransformer	64.90 ± 0.25	62.53 ± 0.27	62.21 ± 0.26	62.25 ± 0.33	81.52 ± 0.29	68.87 ± 0.49
	PatchTST	66.26 ± 0.40	65.08 ± 0.41	64.97 ± 0.51	64.95 ± 0.42	83.07 ± 0.45	71.70 ± 0.61
	FEDformer	77.63 ± 2.37	76.76 ± 2.17	76.68 ± 2.48	76.60 ± 2.46	91.67 ± 1.34	84.94 ± 2.11
	CrossGNN	95.88 ± 0.41	95.74 ± 0.43	95.69 ± 0.46	95.71 ± 0.44	99.21 ± 0.10	98.97 ± 0.14
	FourierGNN	71.42 ± 1.18	69.85 ± 1.26	69.37 ± 1.11	69.51 ± 1.17	86.02 ± 0.92	78.44 ± 1.35
	TodyNet	96.34 ± 0.37	96.21 ± 0.35	96.17 ± 0.39	96.18 ± 0.36	99.46 ± 0.07	99.18 ± 0.09

Empty cells indicate that the corresponding method was not evaluated under that dataset. Red and blue values indicate the best and second-best results, respectively, for each metric within each dataset.

### 3.4 Results of subject-independent

Table 4 presents the experimental results on the three EEG datasets under the Subject-Independent setting. Overall, ScaleSpecter achieves the best or near-best numerical performance on ADFTD, APAVA, and TDBrain, suggesting favorable cross-subject generalization ability.

**Table 4:** Performance comparison of models on EEG datasets.

Datasets	Models	Accuracy	Precision	Recall	F1 score	AUROC	AUPRC
**APAVA (2-Classes)**	ScaleSpecter	80.94 ± 1.06	82.86 ± 1.92	77.19 ± 1.85	78.10 ± 1.84	82.87 ± 3.22	85.82 ± 3.22
	KEMed	78.74 ± 1.48	83.17 ± 2.06	72.15 ± 2.22	72.58 ± 2.58	88.54 ± 1.18	88.68 ± 1.09
	GPT4TS	77.78 ± 4.23	79.36 ± 5.67	75.36 ± 3.22	76.00 ± 3.71	85.55 ± 4.64	85.62 ± 5.09
	Medformer	76.32 ± 1.99	77.89 ± 3.50	73.37 ± 1.50	71.09 ± 6.88	81.96 ± 1.32	82.75 ± 1.28
	iTransformer	74.55 ± 1.66	74.77 ± 2.10	71.76 ± 1.72	72.30 ± 1.79	85.59 ± 1.55	84.39 ± 1.57
	PatchTST	67.38 ± 1.83	78.31 ± 1.59	60.41 ± 2.30	56.81 ± 3.60	65.20 ± 4.30	67.61 ± 3.76
	FEDformer	74.49 ± 2.08	74.34 ± 1.31	73.19 ± 3.56	73.01 ± 3.43	83.65 ± 1.96	82.86 ± 2.29
	CrossGNN	75.22 ± 2.12	80.59 ± 3.31	70.61 ± 2.46	70.89 ± 2.85	73.06 ± 5.30	73.27 ± 4.86
	FourierGNN	57.25 ± 2.05	55.03 ± 1.70	54.55 ± 1.42	54.27 ± 1.51	56.43 ± 2.13	55.21 ± 1.72
	TodyNet	73.05 ± 4.57	75.26 ± 7.06	71.06 ± 2.86	71.05 ± 3.34	77.52 ± 5.97	78.44 ± 5.24
	EEGNet	62.24 ± 14.30	60.66 ± 19.54	58.42 ± 14.62	57.87 ± 16.08	63.73 ± 19.65	64.46 ± 19.14
	EEGInception	71.19 ± 7.57	76.32 ± 9.09	65.52 ± 9.62	62.92 ± 14.98	68.95 ± 9.61	70.24 ± 9.96
	EEGConformer	73.74 ± 3.09	77.51 ± 4.84	69.40 ± 3.86	69.47 ± 4.59	82.13 ± 3.82	82.01 ± 4.23
**TDBRAIN (2-Classes)**	ScaleSpecter	91.02 ± 0.72	91.13 ± 0.77	91.02 ± 0.72	91.01 ± 0.73	97.60 ± 0.44	97.68 ± 0.44
	KEMed	87.29 ± 2.77	87.46 ± 2.65	87.29 ± 2.77	87.28 ± 2.78	95.25 ± 2.37	95.39 ± 2.44
	GPT4TS	86.90 ± 2.35	86.91 ± 2.33	86.90 ± 2.35	86.89 ± 2.35	94.55 ± 1.50	94.71 ± 1.46
	Medformer	87.11 ± 0.67	87.18 ± 0.67	87.21 ± 0.67	86.81 ± 0.67	95.67 ± 0.06	95.23 ± 0.08
	iTransformer	74.73 ± 1.05	74.78 ± 1.04	74.73 ± 1.05	74.72 ± 1.05	83.38 ± 1.14	83.76 ± 1.29
	PatchTST	78.54 ± 5.16	78.76 ± 5.31	78.54 ± 5.16	78.51 ± 5.15	86.17 ± 5.46	83.75 ± 7.23
	FEDformer	77.67 ± 1.45	78.15 ± 1.82	77.67 ± 1.45	77.58 ± 1.41	86.63 ± 1.40	86.71 ± 1.54
	CrossGNN	82.40 ± 2.93	82.59 ± 2.95	82.40 ± 2.93	82.37 ± 2.94	91.86 ± 2.24	92.18 ± 2.20
	FourierGNN	67.10 ± 1.40	67.18 ± 1.45	67.10 ± 1.40	67.07 ± 1.37	73.21 ± 1.50	71.40 ± 1.44
	TodyNet	86.85 ± 2.31	87.04 ± 2.17	86.85 ± 2.31	86.85 ± 2.31	89.79 ± 0.33	94.80 ± 0.31
	EEGNet	80.06 ± 7.76	81.07 ± 6.31	80.06 ± 7.76	79.73 ± 8.33	88.58 ± 7.48	88.73 ± 7.73
	EEGInception	88.37 ± 3.00	89.17 ± 2.64	88.37 ± 3.00	88.31 ± 3.04	95.25 ± 2.43	95.36 ± 2.43
	EEGConformer	87.31 ± 1.73	87.53 ± 1.82	87.31 ± 1.73	87.30 ± 1.72	95.73 ± 0.57	95.76 ± 0.44
**ADFTD (3-Classes)**	ScaleSpecter	54.43 ± 1.01	52.26 ± 1.09	50.20 ± 1.01	50.48 ± 1.05	71.00 ± 1.24	52.15 ± 1.78
	KEMed	51.85 ± 2.92	48.75 ± 1.11	48.25 ± 1.37	47.66 ± 1.10	68.19 ± 0.92	51.06 ± 1.30
	GPT4TS	54.11 ± 0.67	47.76 ± 0.94	46.63 ± 0.87	44.66 ± 0.58	67.86 ± 0.52	49.97 ± 0.67
	Medformer	53.17 ± 0.57	52.86 ± 2.94	44.08 ± 0.42	40.36 ± 0.42	63.60 ± 1.30	46.74 ± 1.10
	iTransformer	52.60 ± 1.59	46.78 ± 1.27	47.27 ± 1.29	46.79 ± 1.13	67.26 ± 1.16	49.53 ± 1.21
	PatchTST	43.97 ± 1.63	42.14 ± 1.46	41.36 ± 0.85	41.26 ± 1.16	59.29 ± 1.04	41.49 ± 1.32
	FEDformer	46.19 ± 0.84	46.12 ± 1.63	44.04 ± 1.50	43.57 ± 1.43	62.27 ± 1.51	45.67 ± 1.44
	CrossGNN	51.24 ± 1.96	46.21 ± 1.25	46.48 ± 1.42	46.07 ± 1.26	66.63 ± 1.80	48.65 ± 1.76
	FourierGNN	49.95 ± 0.92	43.15 ± 1.57	44.51 ± 1.17	43.59 ± 1.33	62.39 ± 1.49	45.60 ± 1.18
	TodyNet	45.18 ± 2.29	43.82 ± 2.50	42.48 ± 2.24	42.22 ± 2.73	61.08 ± 2.64	43.82 ± 2.35
	EEGNet	52.40 ± 3.35	50.41 ± 5.53	47.26 ± 3.24	45.85 ± 3.38	67.73 ± 3.88	50.47 ± 4.38
	EEGInception	51.28 ± 1.74	50.21 ± 3.16	46.53 ± 1.27	45.91 ± 1.76	66.08 ± 2.13	49.54 ± 2.25
	EEGConformer	53.56 ± 2.05	49.00 ± 2.29	48.17 ± 1.57	46.81 ± 1.27	68.86 ± 1.63	50.69 ± 2.30

Red and blue values indicate the best and second-best results, respectively, for each metric within each dataset.

On the ADFTD dataset, ScaleSpecter achieves leading results on key metrics, including Accuracy, Recall, F1-score, AUROC, and AUPRC, indicating that the combination of multiscale temporal fusion and frequency-domain modeling can improve model robustness in a three-class EEG classification scenario characterized by low signal-to-noise ratio and complex class boundaries.

On the APAVA dataset, ScaleSpecter achieves the highest numerical Accuracy and F1-score, indicating favorable discriminative capability under the adopted split. This result suggests that, in Alzheimer's disease EEG recognition tasks with more pronounced cross-subject distribution shifts, the proposed frequency-domain-guided cross-scale fusion mechanism still delivers gains.

On the TDBrain dataset, ScaleSpecter achieves the best results in terms of F1-score, AUROC, and AUPRC, indicating that it can effectively capture multiscale dependencies in EEG signals with a larger number of channels and more complex structures. Taken together, the results across the three datasets show that ScaleSpecter generally achieves competitive or better numerical results than existing single-scale Transformer-based and frequency-domain modeling baselines in classification accuracy, feature representation capability, and cross-subject robustness.

To further examine whether the numerical improvements under the subject-independent setting are stable across repeated runs, we conducted paired statistical comparisons between ScaleSpecter and MedFormer, which is the closest reproducible medical time-series Transformer baseline. As shown in Table 5, ScaleSpecter shows consistent positive differences on TDBrain across all six metrics and on ADFTD for several key metrics, including Recall, F1-score, AUROC, and AUPRC. In contrast, the statistical evidence on APAVA is weaker, especially for AUROC and AUPRC. These results indicate that the proposed method provides overall and metric-dependent improvements, rather than uniform statistical superiority across all datasets and metrics.

**Table 5:** Raw *p*-values are reported for transparency.

Dataset	Metric	ScaleSpecter	MedFormer	Δ	Wins	Wilcoxon *p*	Paired *t* *p*
APAVA	Accuracy	80.94 ± 1.06	76.32 ± 1.99	+4.62	4/5	0.0625	**0.0457**
APAVA	Precision	82.86 ± 1.92	77.89 ± 3.50	+4.97	4/5	0.0938	0.0705
APAVA	Recall	77.19 ± 1.85	73.37 ± 1.50	+3.82	4/5	0.0625	0.0636
APAVA	F1	78.10 ± 1.84	71.09 ± 6.88	+7.01	4/5	0.0625	0.0628
APAVA	AUROC	82.87 ± 3.22	81.96 ± 1.32	+0.91	2/5	0.5938	0.4436
APAVA	AUPRC	85.82 ± 3.22	82.75 ± 1.28	+3.07	3/5	0.5000	0.3842
TDBRAIN	Accuracy	**91.02** **±0.72**	87.11 ± 0.67	+3.91	5/5	**0.0313**	**0.0106**
TDBRAIN	Precision	**91.13** **±0.77**	87.18 ± 0.67	+3.95	5/5	**0.0313**	**0.0089**
TDBRAIN	Recall	**91.02** **±0.72**	87.21 ± 0.67	+3.81	5/5	**0.0313**	**0.0106**
TDBRAIN	F1	**91.01** **±0.73**	86.81 ± 0.67	+4.20	5/5	**0.0313**	**0.0107**
TDBRAIN	AUROC	**97.60** **±0.44**	95.67 ± 0.06	+1.93	5/5	**0.0313**	**0.0094**
TDBRAIN	AUPRC	**97.68** **±0.44**	95.23 ± 0.08	+2.45	5/5	**0.0313**	**0.0081**
ADFTD	Accuracy	54.43 ± 1.01	53.17 ± 0.57	+1.26	4/5	0.0938	0.1176
ADFTD	Precision	52.26 ± 1.09	52.86 ± 2.94	-0.60	2/5	0.4063	0.1736
ADFTD	Recall	**50.20** **±1.01**	44.08 ± 0.42	+6.12	5/5	**0.0313**	**0.0023**
ADFTD	F1	**50.48** **±1.05**	40.36 ± 0.42	+10.12	5/5	**0.0313**	**0.0005**
ADFTD	AUROC	**71.00** **±1.24**	63.60 ± 1.30	+7.40	5/5	**0.0313**	**0.0008**
ADFTD	AUPRC	**52.15** **±1.78**	46.74 ± 1.10	+5.41	5/5	**0.0313**	**0.0019**

Because multiple metrics are evaluated for each dataset, the results are interpreted with Holm–Bonferroni correction and should be regarded as metric-dependent statistical evidence rather than uniform statistical superiority. Bold *p*-values indicate raw p-values below 0.05. Bold values in the ScaleSpecter column indicate metrics for which ScaleSpecter outperformed MedFormer with a raw Wilcoxon signed-rank test *p*-value below 0.05.

### 3.5 Visualization analysis

**Figure 5:**
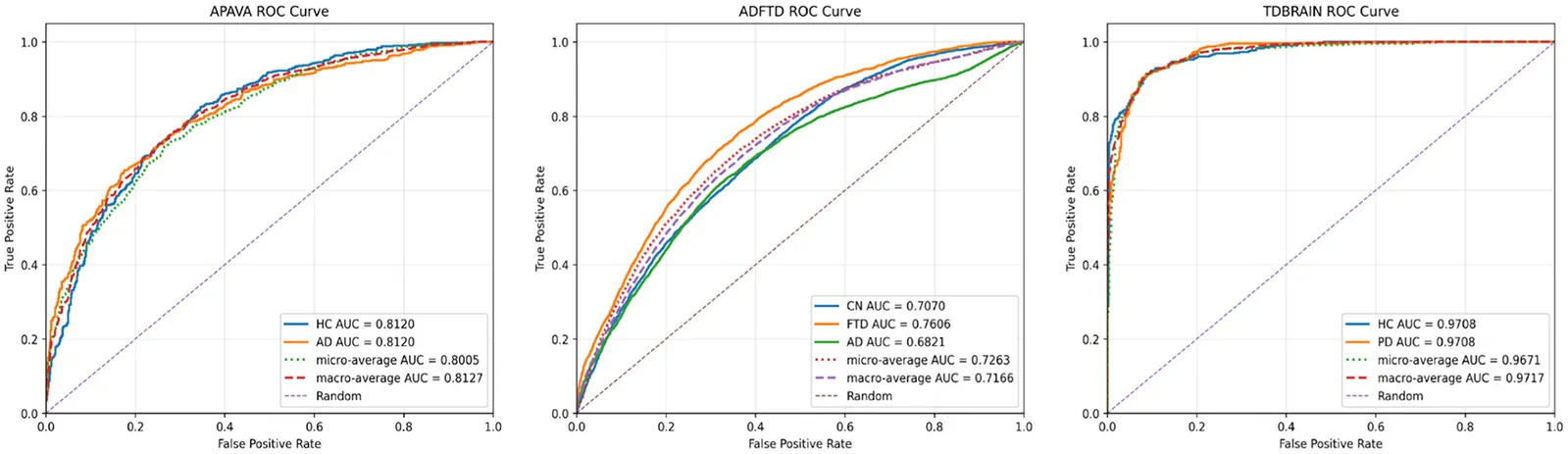
ROC curves on APAVA, ADFTD, and TDBRAIN. The model shows the strongest separability on TDBRAIN, moderate discrimination on ADFTD, and stable binary classification performance on APAVA. The dashed diagonal line denotes the random classifier baseline.

**Figure 6:**
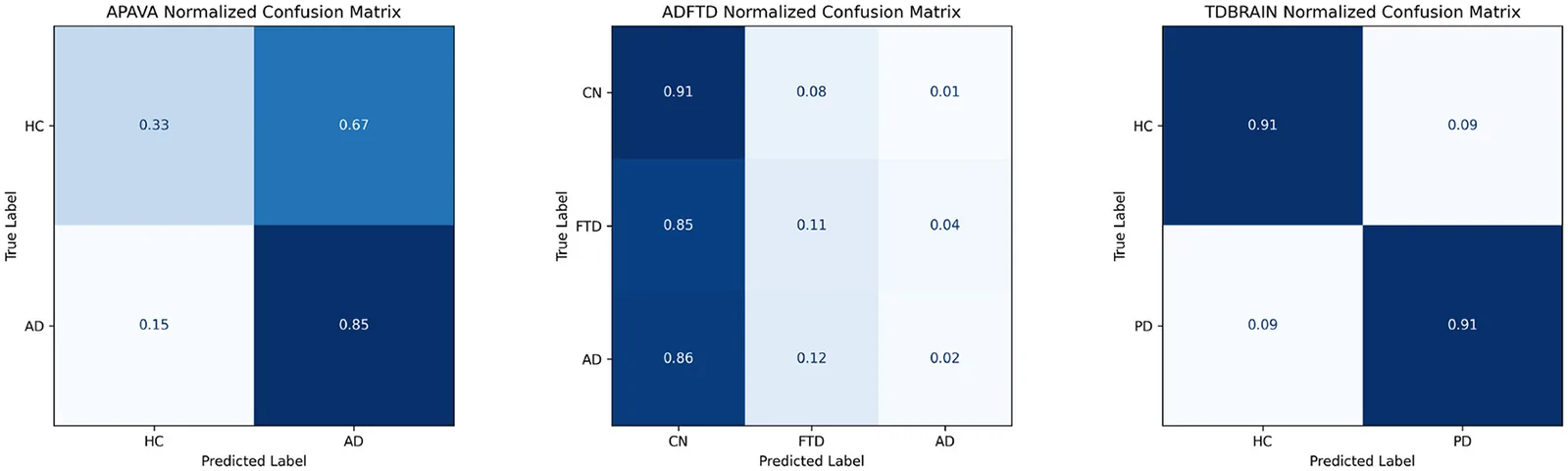
Normalized confusion matrices on APAVA, ADFTD, and TDBRAIN. Rows denote true labels and columns denote predicted labels. TDBRAIN shows balanced HC–PD recognition, while ADFTD exhibits stronger inter-class confusion among CN, FTD, and AD.

To further evaluate the diagnostic behavior of the proposed model, we visualize the receiver operating characteristic (ROC) curves and normalized confusion matrices on APAVA, ADFTD, and TDBRAIN, as shown in Figures 5, 6. Overall, the ROC curves show that the model achieves the strongest discriminative performance on TDBRAIN, with a macro-average AUC of 0.9717 and class-wise AUCs of 0.9708 for both HC and PD. This indicates that the model can reliably rank positive and negative samples on this dataset.

On APAVA, the model also exhibits favorable discriminative ability, achieving a macro-average AUC of 0.8127. The normalized confusion matrix further shows that 85% of AD samples are correctly identified, while 67% of HC samples are misclassified as AD. This suggests that the model tends to make conservative positive predictions on APAVA, yielding high sensitivity to AD cases but relatively lower specificity for HC samples.

Compared with the two binary datasets, ADFTD is a more challenging three-class classification task involving CN, FTD, and AD. Although the hard-label classification accuracy is relatively modest, the ROC curves remain consistently above the random baseline, with a macro-average AUC of 0.7166. Among the three classes, FTD obtains the highest AUC of 0.7606, while AD shows a lower AUC of 0.6921. This indicates that the model still captures non-random disease-related probability rankings, even though the final argmax decision is affected by substantial inter-class ambiguity. The confusion matrix also confirms this challenge, where FTD and AD samples are frequently confused with CN. These observations suggest that ADFTD presents stronger class overlap and remains more difficult for hard-label classification.

### 3.6 Five-fold subject-level cross-validation

Table 6 reports the results of subject-level five-fold cross-validation on the three EEG datasets. Overall, ScaleSpecter maintains competitive and generally top-tier numerical performance under this stricter evaluation protocol, suggesting that its advantage is not solely tied to a single predefined subject split. On ADFTD, ScaleSpecter achieves the highest Accuracy and Recall, while remaining competitive on F1-score, AUROC, and AUPRC, indicating its stable discriminative ability in the three-class EEG classification task. On APAVA, ScaleSpecter achieves the best Precision, Recall, and F1-score among the reproducible baselines, while some ranking-based metrics such as AUROC and AUPRC are slightly higher for several competing methods. This suggests that classification-threshold metrics and ranking-based metrics may respond differently under small-subject cross-validation. On TDBrain, ScaleSpecter achieves the best or near-best performance across the six metrics, leading on most metrics and remaining competitive in Precision, further supporting its robustness on EEG data with more channels and complex subject-level variability.

**Table 6:** 5-fold test results on ADFTD, APAVA, and TDBRAIN datasets.

Dataset	Model	Accuracy	Precision	Recall	F1-score	AUROC	AUPRC
ADFTD	ScaleSpecter	53.28 ± 3.31	51.22 ± 9.78	51.55 ± 8.35	50.13 ± 9.50	69.87 ± 6.36	51.98 ± 7.92
	MedFormer	50.29 ± 3.08	48.45 ± 7.83	47.39 ± 7.75	46.18 ± 8.58	65.50 ± 7.50	49.94 ± 7.43
	iTransformer	46.28 ± 7.10	35.45 ± 10.82	41.76 ± 8.07	35.65 ± 11.62	58.30 ± 8.39	40.33 ± 7.15
	PatchTST	46.97 ± 4.74	46.42 ± 2.40	45.51 ± 2.38	42.93 ± 4.51	63.91 ± 3.67	47.70 ± 2.77
	FEDformer	42.75 ± 3.36	46.08 ± 9.34	43.21 ± 4.19	37.23 ± 1.81	62.64 ± 2.97	44.84 ± 1.77
	CrossGNN	36.79 ± 7.59	28.79 ± 5.86	33.43 ± 0.18	24.52 ± 5.68	49.56 ± 1.32	33.41 ± 0.31
	FourierGNN	53.09 ± 5.02	51.07 ± 3.71	51.37 ± 4.42	50.67 ± 4.44	70.12 ± 4.40	52.18 ± 4.00
	TodyNet	47.81 ± 5.56	47.25 ± 4.75	46.59 ± 4.94	44.71 ± 6.55	64.89 ± 4.03	49.01 ± 4.59
	EEGNet	52.43 ± 7.82	50.32 ± 6.03	50.11 ± 5.67	47.86 ± 6.46	68.86 ± 6.85	52.23 ± 6.04
	EEGInception	49.01 ± 5.12	48.17 ± 4.32	47.97 ± 4.82	45.49 ± 5.54	66.72 ± 5.17	50.08 ± 3.28
	EEGConformer	53.16 ± 6.63	51.23 ± 5.73	51.00 ± 5.51	49.11 ± 7.28	68.41 ± 4.17	52.31 ± 4.73
APAVA	ScaleSpecter	76.82 ± 13.09	76.81 ± 13.51	75.36 ± 13.39	75.55 ± 14.01	82.94 ± 11.82	84.93 ± 10.15
	MedFormer	71.73 ± 16.80	71.66 ± 16.74	72.59 ± 16.88	71.18 ± 16.65	76.16 ± 19.82	80.46 ± 17.99
	iTransformer	76.97 ± 14.67	76.69 ± 16.28	75.35 ± 15.73	74.77 ± 16.51	84.00 ± 14.40	89.59 ± 10.61
	PatchTST	67.49 ± 17.51	68.28 ± 19.33	64.30 ± 16.59	64.09 ± 17.26	71.86 ± 22.45	77.55 ± 19.16
	FEDformer	69.84 ± 21.35	70.26 ± 22.01	70.31 ± 22.58	68.68 ± 21.37	77.51 ± 30.24	82.93 ± 24.39
	CrossGNN	75.86 ± 15.26	75.16 ± 17.52	73.62 ± 16.43	73.26 ± 17.52	83.02 ± 15.15	88.91 ± 10.92
	FourierGNN	67.37 ± 20.68	66.94 ± 20.58	67.25 ± 20.61	66.46 ± 20.25	70.96 ± 26.61	77.87 ± 21.49
	TodyNet	65.24 ± 15.63	63.84 ± 19.53	59.65 ± 14.16	58.18 ± 16.45	71.50 ± 26.65	78.60 ± 22.58
	EEGNet	71.97 ± 7.81	71.57 ± 11.42	68.99 ± 10.88	68.44 ± 10.86	82.93 ± 9.05	79.18 ± 6.22
	EEGInception	70.86 ± 8.31	68.93 ± 21.03	63.15 ± 12.30	59.91 ± 18.73	85.80 ± 15.51	87.98 ± 16.01
	EEGConformer	74.43 ± 18.00	75.59 ± 18.89	73.01 ± 16.74	72.09 ± 18.64	76.80 ± 21.57	78.42 ± 17.78
TDBrain	ScaleSpecter	85.95 ± 7.08	85.60 ± 3.88	84.96 ± 8.12	84.41 ± 9.03	93.36 ± 5.91	90.76 ± 5.52
	MedFormer	84.19 ± 6.03	84.65 ± 4.81	82.09 ± 8.16	82.09 ± 7.06	86.83 ± 8.52	81.80 ± 7.06
	iTransformer	74.78 ± 7.97	73.46 ± 6.71	71.50 ± 9.04	71.08 ± 9.62	79.64 ± 12.68	72.31 ± 10.75
	PatchTST	81.56 ± 8.82	83.55 ± 7.09	77.18 ± 11.29	77.56 ± 11.56	86.37 ± 8.67	80.22 ± 11.22
	FEDformer	78.58 ± 4.10	77.42 ± 3.25	77.25 ± 5.92	76.57 ± 4.70	87.43 ± 5.21	82.16 ± 8.26
	CrossGNN	74.89 ± 8.33	73.34 ± 7.75	71.84 ± 8.72	71.75 ± 9.02	80.05 ± 10.86	70.89 ± 10.20
	FourierGNN	81.61 ± 6.13	81.76 ± 4.71	79.08 ± 8.49	78.96 ± 7.54	90.66 ± 6.08	85.86 ± 9.17
	TodyNet	83.92 ± 7.05	85.80 ± 3.43	81.77 ± 9.07	81.48 ± 8.59	93.09 ± 6.27	90.59 ± 6.30
	EEGNet	79.17 ± 8.98	79.11 ± 7.92	78.75 ± 9.79	77.50 ± 9.34	86.83 ± 12.21	81.17 ± 13.03
	EEGInception	79.47 ± 7.21	79.93 ± 5.72	76.44 ± 9.32	76.20 ± 9.11	87.71 ± 6.86	78.84 ± 6.01
	EEGConformer	83.01 ± 10.33	85.50 ± 7.59	81.91 ± 11.70	80.73 ± 12.27	90.09 ± 9.08	86.72 ± 9.64

Results are reported as mean ± std (%).

It is also worth noting that the standard deviations under five-fold cross-validation are generally larger than those under the fixed-split setting, especially on APAVA. This is expected because each fold contains a limited number of subjects, and the evaluation becomes more sensitive to inter-subject heterogeneity.

### 3.7 Ablation study

To validate the contributions of the key components of ScaleSpecter, systematic ablation studies were conducted on three EEG datasets. Specifically, the following four core modules were removed individually:

- W/O Multi-Scale Patch Embedding: the MSPE module was removed;
- W/O Cross-Scale Attention: the CSA module was removed;
- W/O Spectral Self-Attention: the SSA module was removed and replaced with standard multi-head self-attention;
- W/O Gate Module: the complex-valued gating mechanism in SSA was removed.

Table 7 presents the ablation results obtained on multiple datasets to evaluate the effectiveness of the key modules of the proposed model, namely MSPE, CSA, SSA, and Gate. The results show that the full model (FULL) achieves the best or near-best performance across all datasets, indicating that each component plays an important role in the overall architecture.

**Table 7:** Ablation study of ScaleSpecter on EEG datasets.

Dataset	Model	Accuracy	Precision	Recall	F1-score	AUROC	AUPRC
APAVA (2-Classes)	FULL	80.32 ± 1.50	82.86 ± 1.92	77.19 ± 1.85	78.10 ± 1.84	82.87 ± 3.22	85.82 ± 3.22
	w/o MSPE	75.95 ± 3.05	79.38 ± 2.87	72.01 ± 4.03	72.38 ± 5.02	72.37 ± 4.71	72.66 ± 4.16
	w/o CSA	71.39 ± 5.13	81.43 ± 2.25	65.29 ± 4.65	63.34 ± 8.79	75.91 ± 3.89	77.12 ± 3.16
	w/o SSA	72.30 ± 3.37	76.54 ± 2.80	67.34 ± 5.46	66.93 ± 5.98	76.12 ± 4.20	73.31 ± 5.15
	w/o Gate	71.28 ± 3.76	82.76 ± 1.63	65.01 ± 5.05	63.10 ± 6.77	77.65 ± 2.11	78.67 ± 2.09
ADFTD (3-Classes)	FULL	54.43 ± 1.01	52.64 ± 1.09	50.20 ± 1.01	50.48 ± 1.05	71.00 ± 1.24	52.15 ± 1.78
	w/o MSPE	52.27 ± 1.19	49.87 ± 1.16	47.10 ± 1.42	47.20 ± 1.47	68.87 ± 2.44	49.57 ± 2.29
	w/o CSA	50.94 ± 1.09	47.67 ± 1.20	46.17 ± 0.33	44.71 ± 1.80	67.44 ± 0.96	48.36 ± 1.03
	w/o SSA	52.97 ± 1.64	49.35 ± 2.23	48.29 ± 1.66	47.93 ± 2.07	70.05 ± 2.61	51.25 ± 2.19
	w/o Gate	51.52 ± 1.05	48.67 ± 1.52	47.53 ± 1.45	47.67 ± 1.61	68.46 ± 1.07	48.65 ± 0.83
TDBRAIN (2-Classes)	FULL	91.02 ± 0.81	91.13 ± 0.77	91.02 ± 0.72	91.01 ± 0.73	97.60 ± 0.44	97.68 ± 0.44
	w/o MSPE	81.15 ± 2.05	80.21 ± 1.26	80.15 ± 2.05	79.94 ± 2.30	89.65 ± 0.71	89.74 ± 0.65
	w/o CSA	90.13 ± 2.05	90.63 ± 2.05	90.13 ± 2.05	90.09 ± 2.06	97.24 ± 1.25	97.26 ± 1.20
	w/o SSA	88.81 ± 1.06	88.85 ± 1.06	88.81 ± 1.06	88.81 ± 1.06	96.00 ± 0.48	96.12 ± 0.47
	w/o Gate	86.71 ± 1.39	87.42 ± 2.30	86.71 ± 1.79	86.65 ± 1.81	95.25 ± 1.77	95.33 ± 1.59

Red and blue values indicate the best and second-best results, respectively, for each metric within each dataset.

On the APAVA dataset, the full model achieves an Accuracy of 80.32% and an F1-score of 78.10%, outperforming all variants with any single module removed in terms of the main threshold-based metrics. Among these variants, removing the MSPE module results in the most pronounced performance degradation, with the F1-score dropping to 72.38%, indicating that the multiscale feature extraction mechanism plays a critical role in capturing temporal patterns. The removal of CSA and SSA also leads to performance degradation, further confirming their contributions to feature weighting and discriminative capability enhancement.

On the ADFTD dataset, the full model again outperforms the other settings, achieving an F1-score of 50.48%. Removing either MSPE or CSA leads to a decline of more than 1% in key metrics, and the removal of MSPE has the greatest impact on Recall, suggesting that the multiscale structure helps improve the model's sensitivity to complex temporal features.

On the TDBrain dataset, the FULL model consistently surpasses all other variants across evaluation metrics, with an F1-score of 91.01% and an AUROC of 97.60%. The removal of any module causes performance degradation to varying degrees, and in particular, removing the SSA module reduces the F1-score to 88.98%, highlighting the importance of SSA in neural signal modeling.

Overall, the MSPE module contributes most substantially to the overall performance improvement, while CSA and SSA further enhance the model's feature representation capability, and the Gate mechanism plays a supportive role in optimizing information flow and model stability. The full model generally exhibits the best numerical performance across the evaluated datasets, validating the rationality and complementarity of the proposed module design.

## 4 Discussion

### 4.1 Multi-scale temporal modeling of non-stationary EEG

One of the core challenges in neurodegenerative disease-related EEG analysis is that discriminative patterns are often distributed across multiple temporal resolutions. Local transient fluctuations may be embedded within long-timescale background rhythms, while the overall signal structure is further affected by inter-individual variability, noise perturbations, and non-stationary dynamics. The results of this study indicate that the advantage of ScaleSpecter does not primarily stem from increased model capacity, but from a representation strategy that is better suited to such multi-resolution signal characteristics. By constructing temporal representations at multiple patch scales and organizing their interactions through cross-scale fusion, ScaleSpecter can jointly preserve short-duration local variations and longer-range rhythmic context. This design reduces the risk of relying only on isolated local perturbations or overly smoothed global trends, thereby providing a more balanced representation for non-stationary EEG classification. For cognitive impairment-related tasks such as Alzheimer's disease, this design is consistent with previous findings that disease-related EEG alterations may involve spectral slowing, reduced synchrony, impaired functional connectivity, and abnormal brain network organization (Wiesman et al., 2022; Chetty et al., 2024; Paitel et al., 2025). Therefore, relying solely on a single temporal scale or a single type of frequency-domain statistic may be insufficient to characterize the complex discriminative patterns of neurodegenerative disease-related EEG.

As shown in Table 8, single-scale configurations exhibit clear limitations. Shorter patches are better able to preserve local short-term perturbations, but they provide limited coverage of long-timescale background rhythms. Larger patches include broader temporal context, but may dilute short-duration discriminative patterns, leading to declines in Accuracy and F1-score. Meanwhile, homogeneous multi-patch configurations, in which the same patch length is repeatedly used, do not achieve performance comparable to heterogeneous multi-scale combinations. In contrast, the heterogeneous combination 2, 16, 24, 24 achieves the best overall performance. This suggests that the useful discriminative information in non-stationary EEG is unlikely to be concentrated at a single temporal granularity, but is more likely distributed across both local transient dynamics and slowly varying background activity. In this sense, the role of multi-scale patch representation is not merely to increase the number of tokens, but to provide a candidate representation space that spans different temporal resolutions at the input stage, allowing subsequent cross-scale interactions to be built upon richer temporal structures.

**Table 8:** Patch length study on APAVA (2-class).

Data	Patch setting	Acc (%)	Prec (%)	Rec (%)	F1 (%)	AUROC	AUPRC
APAVA	{2}	76.87 ± 3.68	80.29 ± 2.43	72.86 ± 4.92	73.29 ± 5.71	70.95 ± 3.51	71.36 ± 3.36
	{4}	78.74 ± 0.80	**83.10 ± 1.05**	74.79 ± 1.04	75.69 ± 1.09	76.46 ± 2.95	78.18 ± 2.48
	{8}	76.63 ± 1.57	80.00 ± 2.72	72.87 ± 1.52	73.38 ± 1.58	77.73 ± 3.62	78.65 ± 3.80
	{12}	71.89 ± 4.52	72.68 ± 6.34	68.24 ± 5.00	68.59 ± 5.38	75.48 ± 4.77	75.44 ± 4.90
	{16}	71.09 ± 2.83	71.90 ± 4.62	67.44 ± 2.89	67.68 ± 2.99	78.65 ± 4.03	78.28 ± 4.07
	{24}	74.27 ± 2.41	74.22 ± 2.19	72.93 ± 1.87	72.92 ± 2.22	81.93 ± 2.14	80.91 ± 2.62
	{32}	69.41 ± 2.89	68.95 ± 3.50	66.14 ± 2.90	66.36 ± 3.09	75.93 ± 3.30	74.11 ± 3.70
	{2, 2, 2, 2}	64.67 ± 2.70	69.67 ± 4.30	59.50 ± 6.09	55.53 ± 10.63	66.02 ± 1.12	65.06 ± 1.27
	{4, 4, 4, 4}	75.60 ± 5.18	78.53 ± 7.38	71.67 ± 5.55	72.12 ± 6.55	75.75 ± 5.51	76.84 ± 5.94
	{8, 8, 8, 8}	68.99 ± 2.99	71.46 ± 5.94	66.33 ± 1.71	65.70 ± 1.97	75.38 ± 3.21	76.16 ± 3.32
	{12, 12, 12, 12}	71.66 ± 3.62	72.31 ± 4.08	69.42 ± 2.07	69.50 ± 2.68	77.22 ± 1.42	77.87 ± 1.83
	{16, 16, 16, 16}	72.58 ± 2.52	74.44 ± 3.77	69.30 ± 2.08	69.29 ± 2.25	79.54 ± 2.48	79.60 ± 2.52
	{24, 24, 24, 24}	71.77 ± 1.19	70.79 ± 1.23	70.58 ± 1.41	70.65 ± 1.31	78.07 ± 1.62	77.74 ± 1.56
	{32, 32, 32, 32}	71.80 ± 1.29	71.77 ± 1.70	68.44 ± 1.72	68.76 ± 1.89	77.29 ± 3.29	76.05 ± 3.43
	**{2, 16, 24, 24}**	**80.32 ± 1.50**	82.86 ± 1.92	**77.19 ± 1.85**	**78.10 ± 1.84**	**82.87 ± 3.22**	**85.82 ± 3.22**

Bold values indicate the best result for each evaluation metric among all patch settings.

This observation also provides a signal-level interpretation of the effectiveness of heterogeneous temporal modeling. Neurodegenerative disease-related EEG may contain subtle local fluctuations, changes in background rhythmic activity, and interactions between short- and long-timescale patterns. A single temporal window is unlikely to capture these different types of information simultaneously. By contrast, heterogeneous patch lengths provide complementary temporal views, enabling the model to encode fine-grained local variations and relatively slow temporal dynamics within a unified representation space. The improved performance of the heterogeneous setting therefore supports the necessity of multi-resolution temporal modeling for non-stationary EEG classification.

**Figure 7:**
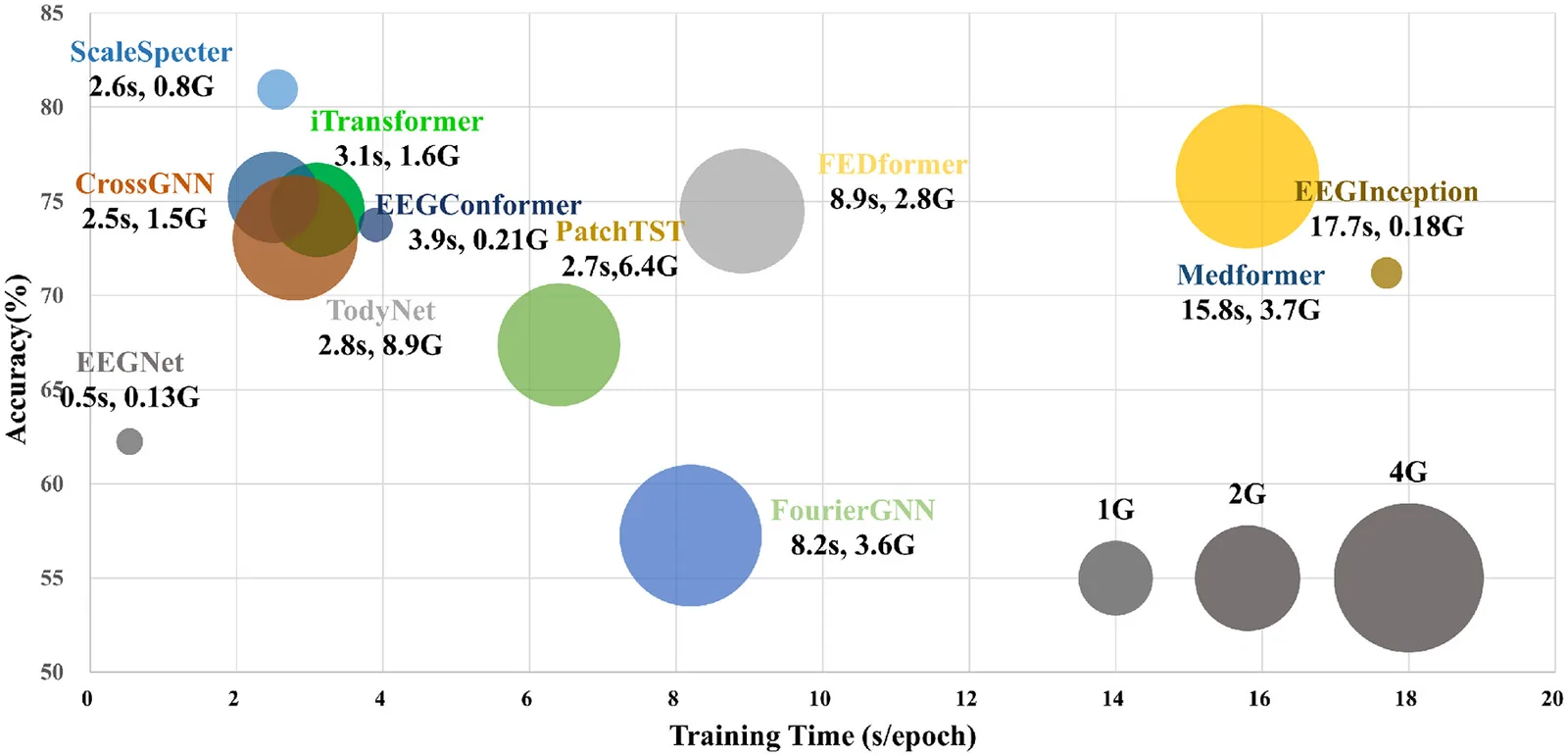
ScaleSpecter achieves high accuracy while maintaining relatively low training time per epoch and memory consumption, demonstrating a favorable performance-efficiency trade-off.

The benefits of this architectural design are achieved without introducing excessive computational overhead. The accuracy-performance distribution shown in Figure 7 indicates that ScaleSpecter maintains high classification accuracy on the APAVA dataset while exhibiting relatively low inference latency and memory consumption, demonstrating a favorable performance-efficiency trade-off. In other words, the performance improvement is not obtained by simply increasing computational cost, but by more effectively organizing temporal, frequency-domain, and cross-scale information. For physiological signals such as EEG, which have potential applications in auxiliary screening and long-term monitoring, this characteristic is important because practical deployment requires models that can reliably extract discriminative patterns under limited computational resources.

### 4.2 Frequency-domain modulation and spectral preference

**Figure 8:**
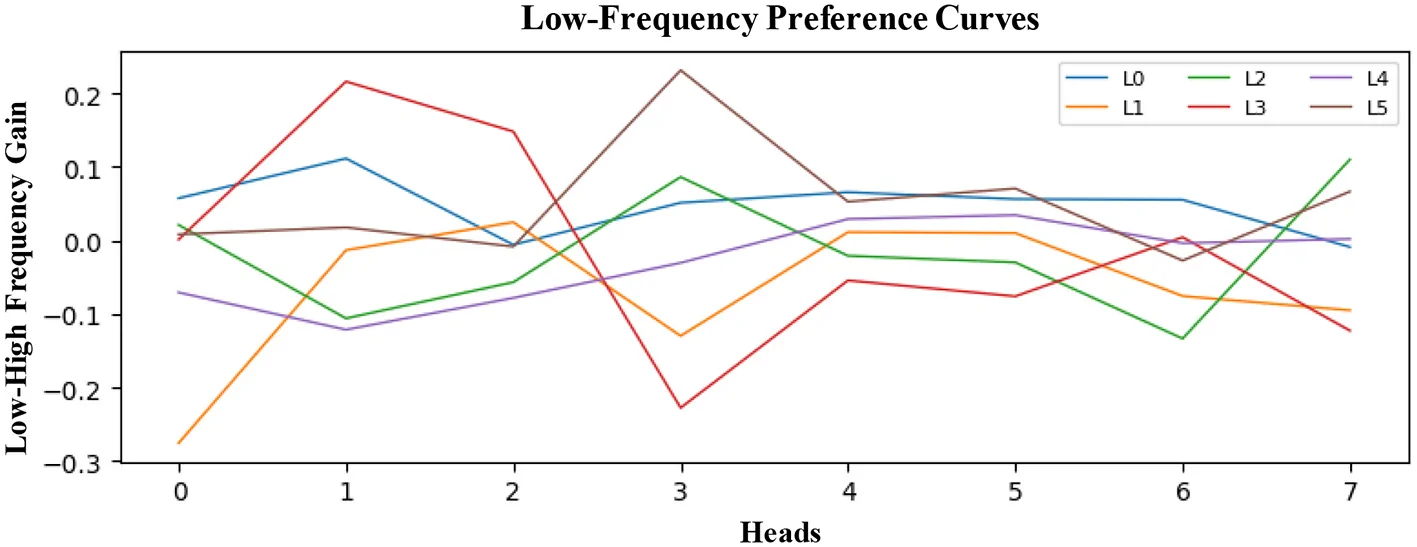
Low-Frequency Preference Curves of the SSA module. The y-axis represents the relative gain of low-frequency components over high-frequency ones, while the x-axis denotes the head index. The divergent curves across Layers 0–5 demonstrate that different attention heads adaptively specialize in capturing distinct frequency bands, ensuring comprehensive spectral feature extraction.

From a frequency-domain perspective, the low-frequency preference curves shown in Figure 8 provide insight into the internal behavior of the SSA module. Compared with early layers, attention heads in deeper layers exhibit more diverse frequency preferences. Some heads maintain stronger responses to low-frequency components, whereas others show relatively higher sensitivity to higher-frequency local variations. This indicates that SSA does not treat all frequency bands uniformly during representation learning, but instead forms differentiated spectral responses across heads and layers.

Such head-wise spectral differentiation is consistent with the complex frequency characteristics of neurodegenerative disease-related EEG. Previous studies have reported that Alzheimer's disease-related EEG is often associated with enhanced low-frequency activity, slowing of background rhythms, and impaired organization of higher-frequency activity (Wiesman et al., 2022; Chetty et al., 2024; Devulder et al., 2025). In this context, the differentiated spectral responses learned by SSA can be regarded as a model-level organization of slowly varying background rhythms and relatively fast local components. Rather than applying a uniform frequency-domain enhancement, SSA adaptively assigns different spectral roles to different heads, allowing the model to represent heterogeneous frequency components within a unified attention structure.

This behavior further supports the contribution of frequency-domain modulation to non-stationary EEG classification. By introducing learnable complex spectral filtering, SSA adjusts the amplitude- and phase-related characteristics of token representations in the frequency domain before constructing head-wise interactions. As a result, the model can preserve low-frequency rhythmic information while maintaining sensitivity to local high-frequency variations. The observed spectral preference therefore suggests that the performance improvement of ScaleSpecter is related not only to the use of frequency-domain information, but also to the differentiated organization of frequency-specific representations.

### 4.3 Cross-scale and spatially structured discriminative patterns

**Figure 9:**
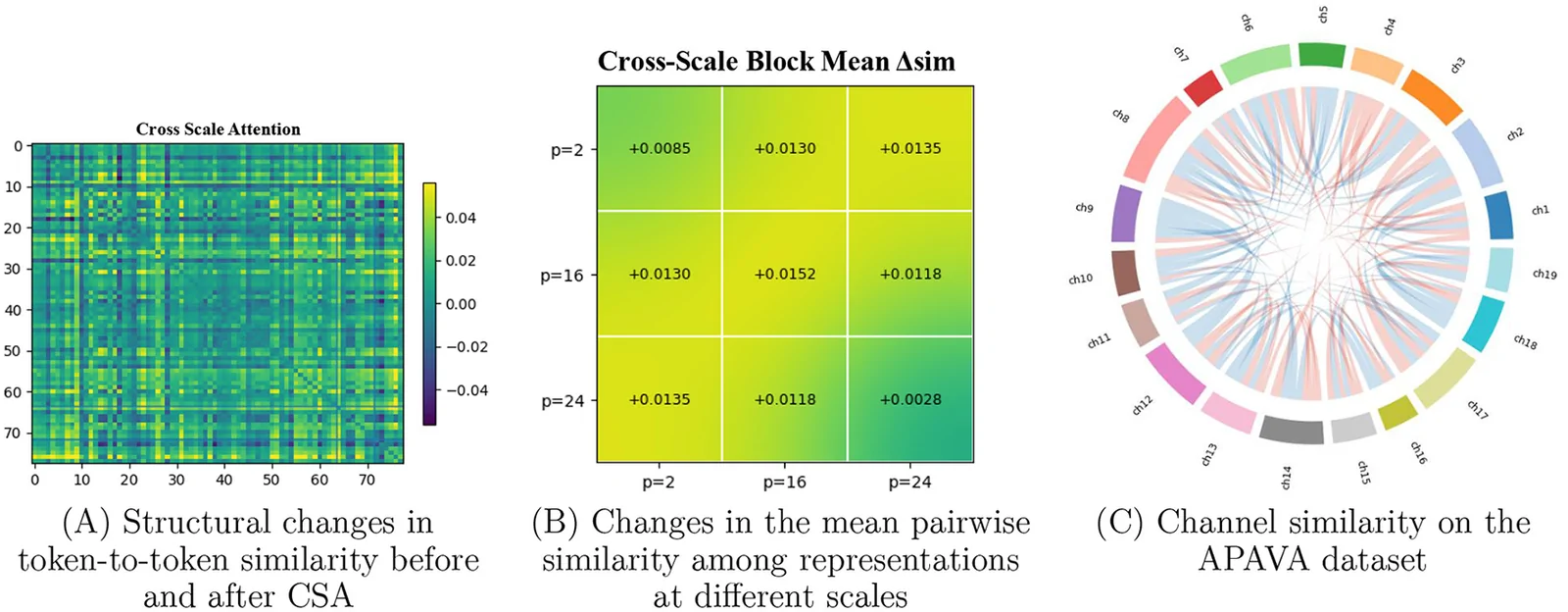
Visualization analysis of the internal representational structure of ScaleSpecter. **(A)** CSA alters the way in which relationships among multiscale tokens are organized, rather than performing globally uniform smoothing; **(B)** the mean similarity among representations at different scales is generally increased, indicating improved cross-scale consistency; **(C)** the channel dependencies learned by the model exhibit a clear structured pattern, indicating that inter-channel collaborative information is preserved. **(A)** Structural changes in token-to-token similarity before and after CSA. **(B)** Changes in the mean pairwise similarity among representations at different scales. **(C)** Channel similarity on the APAVA dataset.

Corresponding to the frequency-domain preferences, the changes in cross-scale relationships further reveal how ScaleSpecter reorganizes multi-scale representations. Figure 9a illustrates the changes in token-to-token cosine similarity before and after the introduction of CSA. The similarity matrix does not show a uniform increase or decrease, but instead presents a structured redistribution pattern. The block-level statistics in Figure 9b further show that positive gains are generally observed among fine-, medium-, and coarse-grained tokens, indicating that CSA enhances the consistency among representations at different temporal scales. Together with the performance degradation observed when CSA is removed in the ablation study, these results suggest that CSA contributes not only to the final classification performance, but also to the organization and calibration of multi-scale representational relationships.

This representational reorganization is important for non-stationary EEG classification, where discriminative patterns are often distributed across different temporal resolutions. Local fluctuations may be embedded within slowly varying background rhythms, and their discriminative value can depend on the broader temporal context. Rather than simply smoothing token representations, CSA allows features at different temporal scales to be updated under a shared contextual structure. In this way, fine-grained and coarse-grained tokens can provide complementary information during representation learning, which helps the model construct more stable multi-scale features.

The same structural property is also reflected in the inter-channel relationships. Figure 9c shows that the channel dependencies learned by the model are not randomly distributed, but exhibit a relatively structured pattern. This indicates that ScaleSpecter does not treat individual EEG channels as completely independent inputs during the discriminative process. Since each patch jointly encodes the multichannel dynamics within the same temporal window, the model can preserve inter-channel collaborative information from the input stage and further organize such relationships in subsequent layers. For multichannel EEG classification, this property is beneficial because disease-related differences may be reflected not only in single-channel temporal patterns, but also in coordinated variations across channels.

**Figure 10:**
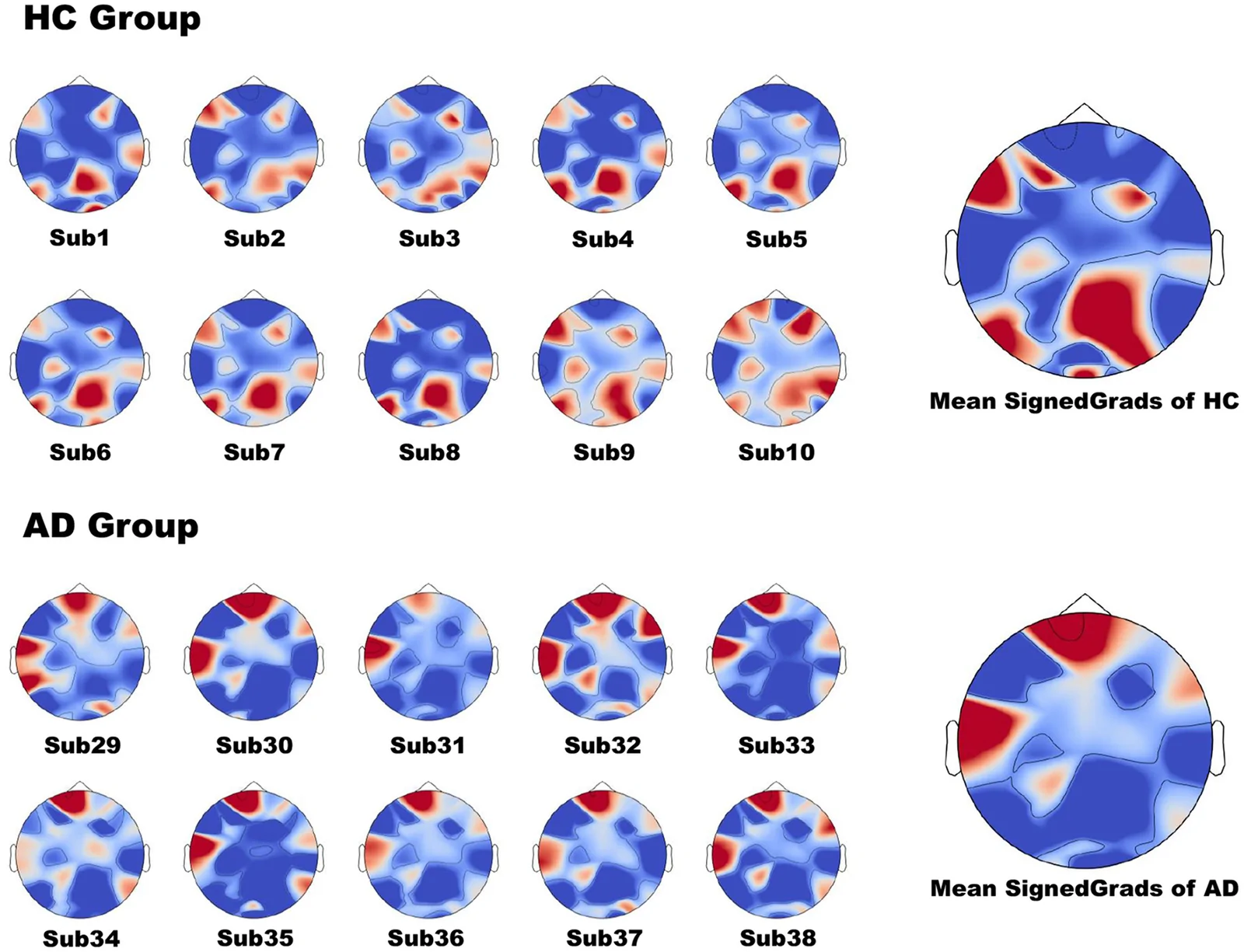
Visualization of the signed gradients for healthy control (HC) and Alzheimer's disease (AD) samples. Red indicates positive gradients that increase the predicted probability of the target class, whereas blue indicates inhibitory effects.

The spatial visualization results provide additional evidence for the structured discriminative patterns learned by the model. As shown in Figure 10, the signed-gradient responses of HC and AD samples are not uniformly distributed across sensors, but show different regional tendencies. The HC group exhibits relatively stronger positive responses over posterior sensors, whereas the AD group shows more prominent responses over frontotemporal and midline sensors, accompanied by weaker posterior responses. Previous studies have reported that Alzheimer's disease-related abnormalities may involve posterior association regions, the medial temporal lobe, and broader networks related to cognitive control and memory integration (Ereira et al., 2024; Seoane et al., 2024; Paitel et al., 2025). The spatial patterns observed in Figure 10 are broadly consistent with these reported regional tendencies, suggesting that the model's discriminative responses contain meaningful spatial structure.

It should be noted that the signed-gradient maps reflect sensor-level model sensitivity rather than direct source-level neural activity. Therefore, the observed spatial distributions should be interpreted as discriminative patterns learned from multichannel EEG signals, rather than as direct evidence of specific neural generators or cognitive-network mechanisms. Within this scope, the visualization results indicate that ScaleSpecter captures spatially organized differences between HC and AD samples, complementing the temporal and frequency-domain evidence provided by the previous analyses.

Taken together, these observations suggest that ScaleSpecter learns discriminative representations from multiple complementary perspectives. Table 8 indicates that discriminative information cannot be adequately captured by a single temporal resolution and benefits from heterogeneous multi-scale representations. Figure 8 shows that SSA forms differentiated spectral preferences across heads and layers, suggesting that different frequency components are organized in a non-uniform manner. Figures 9a, b indicate that CSA changes the relationships among representations at different temporal scales, while Figures 9c, 10 show that the learned discriminative patterns also exhibit spatial and channel-structural properties. These results suggest that the effectiveness of ScaleSpecter arises from the joint organization of multi-scale temporal information, frequency-domain modulation, and multichannel spatial structure, which is consistent with the complex signal characteristics of neurodegenerative disease-related EEG (Paitel et al., 2025; Ereira et al., 2024; Devulder et al., 2025).

### 4.4 Limitations and future work

Several limitations should be noted. The present experiments were conducted on three public EEG datasets, which differ in sample size, acquisition conditions, channel configuration, and preprocessing procedures. Although the results on APAVA, ADFTD, and TDBrain suggest that ScaleSpecter can provide effective representations for different neurodegenerative disease-related EEG classification tasks, the current evidence is still limited by the scale and heterogeneity of the available data. Therefore, the generalization ability of the proposed framework needs to be further examined on larger multicenter cohorts and independent clinical datasets.

Another limitation lies in the evaluation protocol. Although subject-independent settings were adopted to reduce sample-level leakage, part of the evaluation still depends on predefined subject partitions. Such partitions provide a controlled comparison among methods, but they may not fully cover the variability caused by different subject compositions. In future studies, repeated subject-level cross-validation, leave-*N*-subjects-out evaluation, or external validation would provide a more comprehensive estimate of cross-subject robustness. Moreover, as shown by the paired statistical comparison in Table 5, the observed advantages are not uniform across all datasets and metrics. Although ScaleSpecter shows consistent improvements on TDBrain and favorable gains on several ADFTD metrics, the statistical evidence on APAVA is relatively weaker, especially for AUROC and AUPRC. Therefore, the performance gains should be interpreted as overall and metric-dependent improvements rather than universal statistical superiority.

The current study also focuses mainly on disease classification, while more fine-grained clinical questions remain unexplored. Problems such as disease subtyping, progression prediction, severity estimation, and cognitive-dimension modeling may require additional clinical annotations, longitudinal follow-up information, and multimodal biomarkers. Extending ScaleSpecter to these tasks would help determine whether the learned temporal-frequency representations can support more detailed characterization of neurodegenerative processes.

Finally, although the frequency-domain, channel-level, and spatial visualizations provide useful evidence for interpreting the discriminative representations learned by the model, they remain model-derived statistical patterns. The topographic responses are obtained from sensor-level EEG signals and may be affected by volume conduction, reference montage, electrode placement, and channel-level noise. Therefore, these visualizations should not be interpreted as direct evidence of specific neuropathological mechanisms. Future work combining source localization, spatial filtering, longitudinal data, and pathological biomarkers may further clarify the relationship between model-derived discriminative patterns and neurodegenerative changes.

## 5 Conclusion

In this study, we proposed ScaleSpecter, a frequency-aware multiscale framework for neurodegenerative disease-related EEG classification under non-stationary conditions. By combining heterogeneous multiscale temporal representations with frequency-domain amplitude- and phase-aware modulation, ScaleSpecter provides a unified way to model local transient variations, long-timescale rhythmic context, and their cross-scale interactions. Experiments on ADFTD, APAVA, and TDBrain show that the proposed framework achieves competitive overall performance and favorable cross-subject generalization, demonstrating its effectiveness for modeling complex EEG patterns in neurodegenerative disease-related classification tasks. These results suggest that frequency-domain-guided cross-scale temporal fusion can serve as an effective inductive bias for non-stationary EEG representation learning. Future work will further validate the framework on larger multicenter and independent clinical datasets, and extend it to more fine-grained clinical tasks such as disease subtyping, progression prediction, and cognitive-dimension modeling.

## Data Availability

The original contributions presented in the study are included in the article/supplementary material, further inquiries can be directed to the corresponding author.
