# Correction: ScaleSpecter: a frequency-aware multi-scale patch framework for robust physiological classification under non-stationarity

**DOI:** 10.3389/fnhum.2026.1961497

**Published:** 2026-08-19

**Authors:** Frontiers Production Office

**Affiliations:** Frontiers Media SA, Lausanne, Switzerland

**Keywords:** collaborative feature representation, EEG signals, neurodegenerative diseases, phase-amplitude coupling, sensitive pathological features

Reviewer Hemalatha Balan was erroneously assigned affiliation Dr.N.G.P. Institute of Technology, India. This affiliation has now been changed to KIT-Kalaignarkarunanidhi Institute of Technology, India.

The original version of this article has been updated.

